# Tissue engineering driven regeneration in oral squamous cell carcinoma: from biomaterials to precision gene editing

**DOI:** 10.1186/s43046-026-00378-3

**Published:** 2026-06-22

**Authors:** B Deva darshinii, N Subhapradha, S Raghavi, K Anbarasu

**Affiliations:** Saveetha Institute of Medical And Technical Sciences, Chennai, India

**Keywords:** Oral cancer, Tissue engineering, Regenerative medicine, 3D bio printing, Oral tissue regeneration

## Abstract

Oral cancer is a significant global health challenge, ranking as the sixth most prevalent cancer worldwide, with approximately 377,000 new cases diagnosed annually. The high morbidity and mortality rates are largely attributed to tobacco and alcohol use. While conventional treatments such as surgery, radiation, and chemotherapy have improved survival rates, they often lead to unfavourable aesthetic and functional outcomes. Tissue engineering offers a promising alternative, providing regenerative solutions aimed at restoring both oral function and appearance. By integrating biomaterials, biological systems, and engineering principles, tissue engineering enables the creation of functional tissue replacements. The current review examines different t pathways the potential applications of autologous tissue, oral cancer cell lines, CRISPR, gene-editing technologies, and epigenetic modifications for tissue regeneration. Advanced scaffold technologies that mimic the natural extracellular matrix, along with stem cell-based therapies and bioactive molecules, are employed to support tissue growth and differentiation. Mesenchymal stem cells (MSCs) and induced pluripotent stem cells (iPSCs) show significant potential in regenerating hard and soft oral tissues, while also targeting cancer stem cells (CSCs) to prevent recurrence. Furthermore, innovative technologies like 3D bio printing, combined with vascularization strategies, hold promise for developing patient-specific tissue constructs for reconstructive procedures. In conclusion, tissue engineering offers transformative potential for oral cancer treatment, presenting regenerative therapies that can significantly enhance patient outcomes and quality of life.

## Introduction

Oral squamous cell carcinoma (OSCC) represents the predominant malignancy of the oral cavity and continues to pose a significant global healthcare challenge owing to its steadily increasing incidence and mortality rates. Approximately 377,000 new cases of oral cancer are diagnosed annually worldwide, with a markedly higher prevalence observed among populations exposed to establish risk factors such as tobacco smoking, alcohol consumption, betel quid chewing, and human papillomavirus (HPV) infection [1]. More than 90% of oral malignancies are histopathological classified as OSCC, primarily affecting the lips, tongue, buccal mucosa, gingiva, palate, and floor of the mouth. Due to its aggressive and infiltrative nature, OSCC frequently necessitates extensive surgical excision, resulting in substantial loss of oral and maxillofacial tissues and severe impairment of vital physiological functions including mastication, swallowing, phonation, and respiration. Conventional reconstructive procedures, such as skin grafts and free flap surgeries, are routinely employed following tumor resection; however, these approaches are often associated with donor-site morbidity, limited tissue availability, suboptimal tissue integration, and unsatisfactory functional and aesthetic outcomes [1, 2]. Although radiotherapy and chemotherapy remain integral components of contemporary oral cancer management, these therapeutic modalities frequently induce significant collateral damage to adjacent healthy tissues, including the salivary glands, oral mucosa, and osseous structures. Consequently, patients commonly experience debilitating complications such as xerostomia, mucositis, fibrosis, osteoradionecrosis, delayed wound healing, increased susceptibility to infections, and a profound deterioration in overall quality of life [3, 4]. Therefore, there exists an urgent clinical imperative for advanced therapeutic strategies capable of not only eradicating malignant cells but also facilitating the regeneration and functional restoration of oral tissues following oncologic treatment [3]. In recent years, tissue engineering and regenerative medicine have emerged as transformative interdisciplinary approaches with considerable potential to overcome the limitations associated with conventional oral cancer therapies. Tissue engineering integrates biomaterials, scaffolds, stem cells, growth factors, and biologically active molecules to fabricate functional tissue substitutes that closely replicate the structural, biological, and functional characteristics of native oral tissues [3, 5]. Biomimetic scaffolds function as extracellular matrix-like platforms that support cellular adhesion, proliferation, differentiation, angiogenesis, and tissue remodeling, thereby promoting the regeneration of damaged oral and maxillofacial tissues [2, 3]. A wide range of advanced biomaterials, including hydrogels, silicate bio ceramics, halloysite nanotubes, functionalized Nano diamonds, and chitosan-based nanocomposites, have demonstrated remarkable promise in oral and maxillofacial tissue engineering owing to their superior biocompatibility, osteoconductivity, antimicrobial activity, and controlled drug-release capabilities [1–3, 6]. Among these, halloysite nanotubes (HNTs) have attracted substantial scientific interest as multifunctional Nano carriers for personalized oral and maxillofacial applications because of their distinctive tubular nanostructure, high loading efficiency, and sustained release properties. HNT-based scaffolds are capable of delivering therapeutic agents, growth factors, antimicrobial compounds, and anti-cancer drugs directly to tumor or regenerative sites while minimizing systemic toxicity and enhancing therapeutic precision [1]. Similarly, functionalized Nano diamonds exhibit exceptional physicochemical stability, extensive surface area, and tunable surface chemistry, rendering them highly promising candidates for targeted oral cancer therapy and regenerative dentistry applications [6]. Furthermore, recombinant biologics and growth factor-loaded biomaterials have significantly advanced the field of oral tissue regeneration. Bioactive scaffolds incorporating platelet-derived growth factor (PDGF), bone morphogenetic proteins (BMPs), vascular endothelial growth factor (VEGF), fibroblast growth factors (FGFs), and transforming growth factor-β (TGF-β) have demonstrated substantial potential in enhancing angiogenesis, extracellular matrix deposition, osteogenic differentiation, and tissue repair [5, 7]. Chitosan-based thermoresponsive hydrogels have additionally emerged as highly effective multifunctional delivery platforms owing to their biodegradability, inject ability, mucoadhesive properties, and controlled release behavior, thereby improving localized therapeutic efficacy and regenerative outcomes within the oral environment [3, 5]. Stem cell-based regenerative therapies have further expanded the therapeutic horizon of tissue engineering in oral cancer reconstruction. Mesenchymal stem cells (MSCs), oral stem cells, induced pluripotent stem cells (iPSCs), and urine-derived stem cells (UDSCs) possess remarkable self-renewal, multilineage differentiation, immunomodulatory, and regenerative capabilities that facilitate the regeneration of both hard and soft oral tissues following tumor excision [2, 8, 9]. Moreover, the integration of stem cells with biomimetic hydrogels and nanostructured scaffolds has demonstrated enhanced cellular viability, retention, vascularization, and targeted tissue regeneration, thereby substantially improving regenerative efficacy and clinical outcomes [9]. In addition, emerging technologies such as CRISPR/Cas9-mediated gene editing, artificial intelligence (AI)-assisted biomaterial engineering, and exosome-based Nano therapeutics are revolutionizing the field of precision oral oncology. Bioengineered exosomes and nanoparticle-mediated delivery systems have demonstrated the capability to selectively transport chemotherapeutic agents, nucleic acids, and gene-editing molecules directly to tumor tissues while minimizing off-target toxicity and overcoming therapeutic resistance [4]. Notably, plant-derived exosomes and AI-driven Nano therapeutic platforms have exhibited promising anti-cancer activity through modulation of the tumor microenvironment, suppression of metastatic progression, and enhancement of targeted drug delivery efficiency [4]. Collectively, these interdisciplinary advancements underscore the transformative potential of tissue engineering, regenerative medicine, and nanotechnology in the development of highly personalized therapeutic strategies for oral cancer management and oral-maxillofacial reconstruction.

## Pathophysiology of oral squamous cell carcinoma

### Risk factors and molecular alterations

The pathogenesis of oral squamous cell carcinoma (OSCC) is highly complex and involves multifactorial interactions between environmental carcinogens, genetic susceptibility, epigenetic modifications, chronic inflammation, and tumour micro environmental alterations. Tobacco smoking, smokeless tobacco use, alcohol abuse, betel quid chewing, poor oral hygiene, dietary deficiencies, chronic mucosal irritation, and persistent HPV infection are among the major risk factors implicated in OSCC initiation and progression [1, 3]. These carcinogenic stimuli induce oxidative stress, chronic inflammation, DNA damage, and genomic instability, ultimately resulting in malignant transformation of oral epithelial cells. At the molecular level, OSCC development is associated with dysregulation of multiple oncogenic signalling pathways and genetic abnormalities. Mutations in tumour suppressor genes such as TP53 are among the most frequently observed molecular alterations and are strongly associated with impaired apoptosis, defective DNA repair, and uncontrolled cellular proliferation. Overexpression of epidermal growth factor receptor (EGFR), activation of PI3K/Akt/mTOR signaling pathways, cyclin D1 amplification, dysregulation of Wnt/β-catenin pathways, and aberrant NF-κB activation contribute significantly to tumour growth, angiogenesis, invasion, metastasis, and therapeutic resistance [4]. Chronic inflammatory responses further exacerbate tumour progression through the release of pro-inflammatory cytokines, reactive oxygen species (ROS), matrix metalloproteinase (MMPs), and growth factors that promote epithelial-to-mesenchymal transition (EMT), angiogenesis, and extracellular matrix remodelling. The tumour microenvironment (TME), consisting of fibroblasts, immune cells, endothelial cells, extracellular matrix components, and cancer stem cells (CSCs), plays a crucial role in regulating OSCC heterogeneity and disease progression. CSCs possess self-renewal and tumour-initiating capabilities and exhibit remarkable resistance to radiotherapy and chemotherapy, thereby contributing to recurrence and metastasis [9]. Epigenetic dysregulation also plays a critical role in oral carcinogenesis. DNA methylation abnormalities, histone modifications, microRNA dysregulation, and chromatin remodelling influence the expression of genes involved in proliferation, apoptosis, invasion, and immune evasion. Emerging evidence suggests that targeting epigenetic alterations may provide novel therapeutic opportunities for overcoming drug resistance and suppressing tumour recurrence [4]. Nanotechnology-based biomaterials have recently demonstrated substantial potential in modulating these molecular pathways and enhancing targeted oral cancer therapy. Halloysite nanotubes and Nano diamond-based Nano carriers can selectively deliver anti-cancer drugs, nucleic acids, and bioactive molecules to tumour tissues while minimizing systemic toxicity. Their large surface area, tunable surface chemistry, and sustained release behaviour enable precise molecular targeting and improved therapeutic efficacy [1, 6]. Functionalized Nano diamonds can additionally support bone regeneration, angiogenesis, and antimicrobial activity, making them valuable multifunctional platforms for oral and maxillofacial reconstruction following tumour resection [6]. Similarly, chitosan thermoresponsive hydrogels and growth factor-loaded scaffolds have shown promising capabilities in controlling inflammation, enhancing angiogenesis, and stimulating tissue regeneration within the oral cavity. The incorporation of recombinant biologics such as BMPs, VEGF, PDGF, and FGFs into biomaterial scaffolds further improves osteogenic differentiation, extracellular matrix synthesis, and wound healing [3, 5, 7]. Additionally, stem cell-based regenerative strategies are increasingly being explored for personalized oral cancer therapy. Oral stem cells, MSCs, and UDSCs possess immunomodulatory and regenerative capacities that can facilitate tissue repair while potentially modulating tumour-associated inflammation and micro environmental interactions. The integration of stem cells with biomimetic hydrogels and bioengineered scaffolds can significantly enhance targeted tissue regeneration and post-surgical reconstruction [2, 8, 9].

### Tumor microenvironment and cellular heterogeneity

Oral malignancies, particularly oral squamous cell carcinoma (OSCC), are highly heterogeneous in nature, consisting of multiple cell subpopulations with distinct molecular and genetic characteristics. This cellular heterogeneity significantly complicates treatment because different tumor cell populations may respond variably to chemotherapy, radiotherapy, and immunotherapy [10]. Tumor evolution further contributes to this complexity, as cancer cells undergo genetic alterations and epigenetic modifications that enable them to adapt to environmental stressors such as therapeutic interventions and immune surveillance. In OSCC, common molecular abnormalities include p53 mutations, epidermal growth factor receptor (EGFR) overexpression, and alterations in cell cycle regulatory pathways [11]. A critical component of tumor heterogeneity is the presence of cancer stem cells (CSCs), a specialized subpopulation associated with tumor initiation, metastasis, recurrence, and therapeutic resistance. CSCs possess self-renewal capabilities and can regenerate tumors even after apparent tumor eradication. These cells are particularly resistant to chemotherapy and radiotherapy, making them an important therapeutic target within the CHIP model [12]. In addition, the heterogeneous tumor microenvironment (TME) plays a vital role in influencing tumor progression and treatment response. The TME is composed of cancer cells, fibroblasts, endothelial cells, and various immune cell populations, whose interactions can either promote tumor growth or suppress anti-tumor immune responses. The CHIP model emphasizes the role of this complex microenvironment in driving cellular heterogeneity and contributing to treatment resistance in oral cancer [13].

### Epithelial-to-Mesenchymal Transition (EMT)

Phenotypic plasticity refers to the ability of cancer cells to alter their phenotype in response to external stimuli such as therapeutic interventions and immune pressure. This adaptability plays a crucial role in tumor progression, treatment resistance, and disease recurrence in oral malignancies [14]. Through phenotypic alterations, oral cancer cells can survive under adverse conditions and develop mechanisms that promote tumor aggressiveness and therapeutic evasion. One of the major mechanisms associated with phenotypic plasticity is epithelial-to-mesenchymal transition (EMT), a biological process in which epithelial cancer cells acquire mesenchymal characteristics, resulting in enhanced migratory and invasive capabilities. EMT contributes significantly to tumor invasion, metastasis, and disease progression in oral cancer. Moreover, EMT is closely associated with the development of chemo resistance and immunoresistance, making it a critical target in the CHIP model for designing effective targeted therapies [15].Phenotypic plasticity also influences drug response in oral cancer treatment. Cancer cells can dynamically modify their phenotype to resist chemotherapy and radiotherapy. For instance, some tumor cells activate DNA repair pathways to overcome radiation-induced damage, whereas others upregulate ATP-binding cassette (ABC) transporters that actively efflux chemotherapeutic agents, thereby reducing drug efficacy. The CHIP model helps predict how these phenotypic adaptations influence therapeutic outcomes and aids in the identification of potential biomarkers associated with drug resistance [16].

### Epigenetic dysregulation in OSCC

**Fig. 1:**
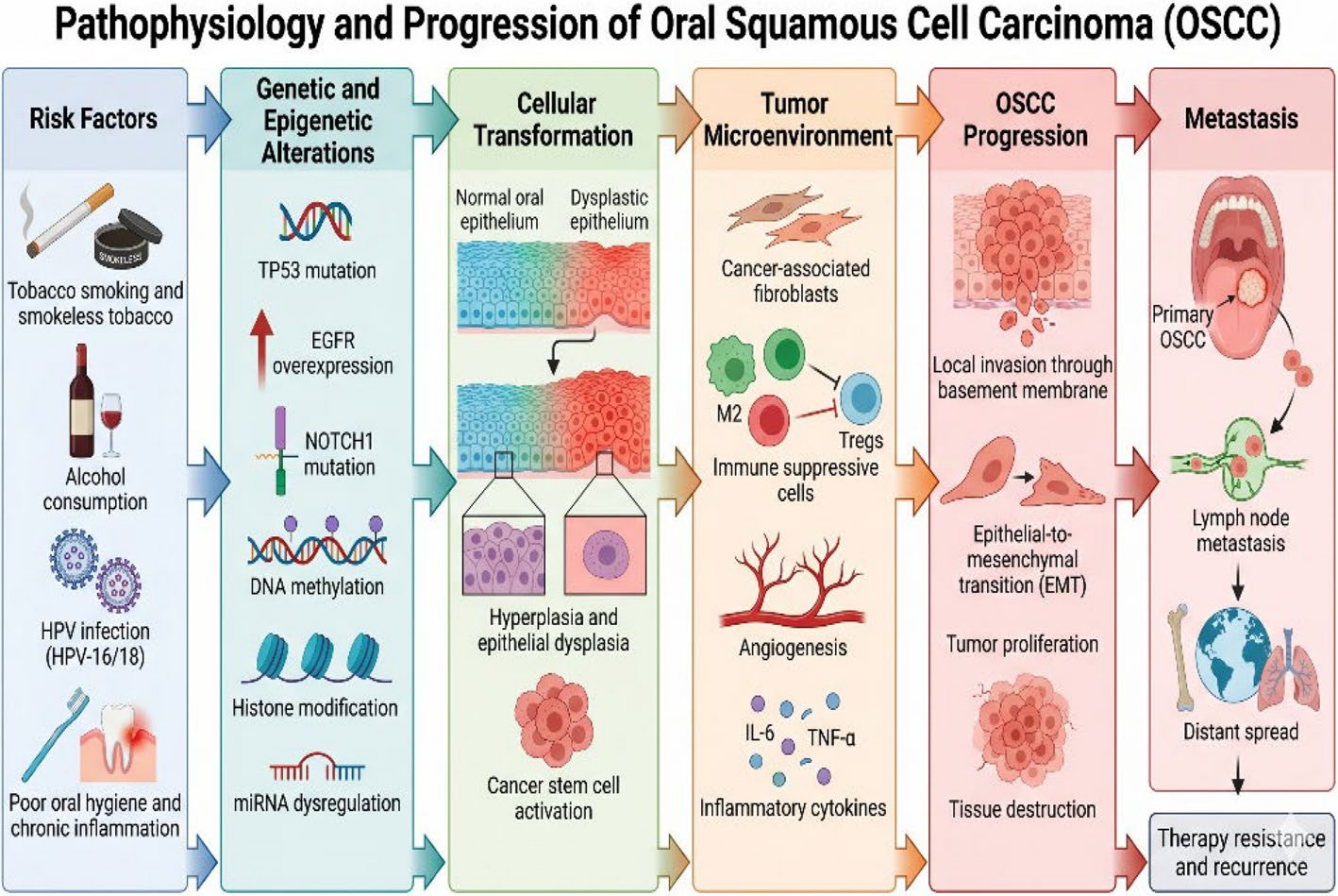
Pathophysiology of OSCC

As an additional strategy to direct genetic engineering methods, epigenetic modifications have become a major focus in the research and treatment of oral cancer. Epigenetic modifications are heritable adjustments in gene expression that do not affect the DNA code itself, in contrast to genetic mutations that change the DNA sequence. These alterations have a major Impact on the development of cancer and are essential in the control of gene activity. By inhibiting tumour suppressor genes or turning oncogenes, aberrant epigenetic changes like DNA methylation and histone modification aid in the development of oral cancer. To undo the epigenetic alterations that encourage the development of cancer, researchers are looking into these modifications as possible therapeutic targets. It explores the different pathways that contribute to oral cancer and how epigenetic treatments may enhance the effectiveness of treatment [17]. One of the most well-studied epigenetic changes is called DNA methylation, and it occurs when a methyl group is added to the cytosine base of DNA, especially in CpG islands, which are areas of the genome that are high in guanine and cytosine nucleotides. Methylation aids in the regulation of gene expression in healthy cells, keeping some genes dormant when not required [18]. Aberrant DNA methylation patterns are frequently found in cases of oral cancer, nevertheless. Tumour suppressor genes, like MLH1 and CDKN2A, are frequently hyper methylated, which silences them. Because of this silencing, vital regulatory controls on cell growth are eliminated, allowing malignant cells to multiply unchecked. Through comprehending the distinct methylation patterns associated with oral cancer, scientists aim to create treatments capable of undoing these modifications [19]. DNA methyl transferase (DNMT) inhibitors are a promising strategy to treat aberrant DNA methylation in oral cancer. Enzymes known as DNMTs catalyse the insertion of methyl groups into DNA, and they are essential for the maintenance of aberrant methylation in cancer cells. Exhibitors that have been demonstrated to reverse DNA hyper methylation and restore the expression of silenced tumour suppressor genes include 5-azacytidine (Azacitidine) and decitabine (5-aza-2'-deoxycytidine). By turning these vital genes back on, DNMT inhibitors have shown in preclinical models of oral cancer that they can slow the growth of tumours. To find out how safe and effective these medications are for patients with oral cancer, clinical trials are currently being conducted [20]. Proteins called histones are the building blocks of chromatin, which encases DNA and forms the skeleton of chromosomes. Histone modifications, including acetylation, methylation, and phosphorylation, affect the degree to which DNA is coiled around these proteins, controlling the expression of genes. The dysregulation of genes involved in cell cycle control, apoptosis, and DNA repair can be caused by aberrant histone modifications in oral cancer. Active transcription of genes is linked to histone acetylation, whereas deacetylation tends to repress gene activity. Tumour suppressor genes may be silenced or oncogenes may be activated because of changes in histone modification patterns in oral cancer, which can accelerate the disease's growth [21]. Gene repression and a more compact chromatin structure are the results of histone deacetylases (HDACs) removing acetyl groups from histones. Tumour suppressor genes are silenced in oral cancer because HDACs are frequently overexpressed in this disease. Histone acetylation can be restored by HDAC inhibitors (HDACis), which reactivates genes that have been silenced. This makes HDACis a promising treatment option for oral cancer. By reactivating tumour suppressor genes and sensitizing cancer cells to chemotherapy and radiation, medications like vorinostat (SAHA) and romidepsin have shown promise in preclinical studies. HDAC inhibitors provide a new way to improve treatment responses for patients with oral cancer by reversing epigenetic silencing [22]. Combining DNMT and HDAC inhibitors with traditional cancer treatments like chemotherapy and radiation therapy is one of the most exciting advances in epigenetic therapy. Treatment sensitivity of a tumour can be influenced by epigenetic changes. For example, epigenetic drugs could reactivate silenced genes involved in DNA repair or apoptosis, increasing the susceptibility of cancer cells to agents that damage DNA, such as radiation or cisplatin. To increase treatment efficacy, decrease tumour resistance, and enhance patient outcomes, clinical trials are investigating the effectiveness of combining DNMT or HDAC inhibitors with conventional oral cancer therapies [23]. MicroRNAs, which are small non-coding RNA molecules, control the expression of genes by directing messenger RNA (mRNA) towards translational repression or degradation. Certain miRNAs function as tumour suppressors or oncogenes in oral cancer and are controlled by epigenetic processes like histone modification and DNA methylation. For instance, silencing of tumour suppressor miRNAs due to hyper methylation of their promoter regions can aid in the development of cancer. On the other hand, epigenetic modifications that encourage the growth of cancer can cause oncogenic miRNAs to be overexpressed. Comprehending the relationship between miRNAs and epigenetic modifications in oral cancer could result in the creation of innovative treatment approaches focused on re-establishing typical miRNA expression patterns [24]. Techniques and mechanisms related to tissue engineering. Despite the remarkable therapeutic potential of CRISPR/Cas9-mediated genome editing and epigenetic modulation in oral squamous cell carcinoma (OSCC), several critical translational and biosafety challenges continue to hinder their widespread clinical implementation [25]. One of the major limitations associated with CRISPR/Cas9 technology is off-target mutagenesis, in which unintended genomic regions may undergo non-specific editing, potentially resulting in genomic instability, activation of oncogenic pathways, or disruption of tumour suppressor genes [26]. Although substantial improvements in guide RNA design and high-fidelity Cas variants have enhanced editing precision, complete elimination of off-target effects remains a significant challenge in clinical oncology applications [27]. Another major obstacle involves the efficient and safe in vivo delivery of CRISPR/Cas9 systems into tumour tissues. Viral delivery vectors, including adenoviral and lentiviral systems, may induce immunogenicity, inflammatory responses, insertional mutagenesis, and cytotoxicity, thereby limiting their long-term clinical safety [26]. Similarly, non-viral nanoparticle-mediated delivery systems often exhibit poor tissue penetration, rapid degradation, inadequate intracellular uptake, and limited retention within the highly dynamic oral microenvironment. The continuous salivary flow, complex vascularization, and heterogeneous stromal architecture of oral tumours further complicate localized delivery efficiency in OSCC therapy [28]. Tumour heterogeneity additionally represents a substantial barrier to precision gene-editing therapies. Distinct OSCC subpopulations frequently exhibit diverse genetic mutations, epigenetic profiles, stemness-associated pathways, and therapeutic sensitivities. Consequently, certain tumour clones may evade CRISPR-mediated targeting or activate compensatory signalling pathways that contribute to therapeutic resistance, tumour recurrence, and metastatic progression. Emerging evidence indicates that cancer stem cells (CSCs) and adaptive tumour micro environmental interactions may further compromise long-term treatment efficacy by promoting survival of resistant cellular populations [27]. Epigenetic therapies also present several unresolved limitations despite their promising therapeutic potential. Current epigenetic editing strategies may induce non-specific chromatin remodelling, unintended gene activation or silencing, and off-target epigenomic modifications that can influence normal cellular homeostasis [29]. Furthermore, the long-term stability, reversibility, and safety of epigenetic modifications remain insufficiently understood, particularly within highly heterogeneous oral tumour microenvironments [30]. The lack of standardized delivery systems and limited clinical validation further restrict the translational applicability of epigenetic therapeutics in oral oncology [31]. In addition to biological and technical limitations, important ethical and regulatory concerns remain associated with genome-editing technologies [25]. Heritable genome modifications, unintended germline alterations, long-term genomic safety, and potential misuse of gene-editing systems continue to generate significant ethical debate within the scientific community [29]. Regulatory approval pathways for CRISPR-mediated therapies also remain highly complex because of concerns regarding patient safety, bio surveillance, and long-term monitoring requirements. Therefore, comprehensive biosafety assessment, precise delivery optimization, and rigorous ethical oversight are essential before large-scale clinical translation of gene-editing and epigenetic therapies can be achieved in oral cancer management [25]. Pathophysiology and Progression of Oral Squamous Cell Carcinoma are summarised in Fig. 1.

## Limitations of contemporary therapeutic approaches

Despite substantial advancements in the diagnosis and management of oral squamous cell carcinoma (OSCC), currently available therapeutic modalities remain associated with significant clinical limitations and long-term complications. Surgical resection continues to represent the primary treatment strategy for OSCC; however, extensive tumor excision frequently results in severe loss of soft and hard oral tissues, leading to profound functional and aesthetic impairments. Essential physiological functions such as mastication, swallowing, speech, and respiration are often compromised following surgery, thereby significantly diminishing patient quality of life [1, 32]. Conventional reconstructive procedures, including auto grafts, allografts, and free flap surgeries, are commonly utilized to restore oral structures; nevertheless, these approaches are associated with donor-site morbidity, limited tissue availability, prolonged surgical duration, poor tissue integration, and inconsistent functional and cosmetic outcomes [32]. Furthermore, the inability of traditional grafting techniques to fully replicate the complex architecture and functionality of native oral tissues remains a major therapeutic challenge [3]. Radiotherapy and chemotherapy, although effective in eliminating malignant cells, are frequently accompanied by substantial collateral damage to adjacent healthy tissues, including the salivary glands, oral mucosa, and osseous structures. Consequently, patients commonly develop severe adverse effects such as xerostomia, mucositis, fibrosis, osteoradionecrosis, dysphagia, impaired wound healing, immunosuppression, and increased susceptibility to secondary infections [3, 4]. In addition, systemic chemotherapy often lacks tumour specificity, resulting in off-target toxicity and reduced therapeutic efficiency. Long-term administration of chemotherapeutic agents may also contribute to multidrug resistance, tumour recurrence, and metastatic progression, thereby limiting overall treatment success [4]. Another major limitation of conventional oral cancer therapies is their inability to adequately address tumour heterogeneity and the complex tumour microenvironment. Oral malignancies are characterized by the presence of diverse cellular subpopulations, including cancer stem cells (CSCs), which possess enhanced self-renewal capacity, therapeutic resistance, and tumour-initiating potential. Conventional therapeutic modalities frequently fail to eradicate CSC populations, thereby contributing to tumour recurrence, invasion, and metastasis [9]. Moreover, chronic inflammation, dysregulated immune responses, and aberrant molecular signalling pathways within the tumour microenvironment further complicate therapeutic efficacy and disease management [4]. Localized drug delivery within the oral cavity also presents considerable challenges because of continuous salivary flow, dynamic mechanical forces, enzymatic degradation, and limited retention of therapeutic agents at the target site. Traditional drug delivery systems often exhibit rapid degradation, poor penetration into tumour tissues, inadequate bioavailability, and uncontrolled release kinetics, thereby reducing therapeutic effectiveness [3]. Although biomaterial-based regenerative approaches have demonstrated promising potential, many currently available scaffolds still suffer from inadequate vascularization, insufficient mechanical stability, uncontrolled biodegradation, and limited long-term clinical validation [2]. Stem cell-based regenerative therapies, despite their considerable promise, are also associated with multiple translational challenges. Issues related to immune compatibility, ethical considerations, potential tumorigenicity, insufficient cell survival, poor engraftment efficiency, and lack of standardized clinical protocols continue to hinder their widespread clinical application [2, 8, 9]. Similarly, advanced nanomaterials such as halloysite nanotubes and functionalized Nano diamonds require further investigation regarding their long-term biosafety, biodegradability, pharmacokinetics, and large-scale manufacturing feasibility before routine clinical implementation can be achieved [1, 6]. Collectively, these limitations underscore the urgent need for innovative multidisciplinary therapeutic strategies capable of integrating tissue engineering, regenerative medicine, nanotechnology, and precision oncology to improve the overall management and reconstruction of oral cancer.

## Overview of revolutionary approaches in the research and development of novel therapeutics for oral squamous cell carcinoma

One of the most promising approaches in tissue engineering for oral cancer management is the utilization of autologous tissues. Since these tissues are derived from the patient’s own body, the risk of immune rejection is significantly reduced, resulting in improved biocompatibility and integration with surrounding tissues. Continuous advancements in the harvesting, manipulation, and implantation of autologous tissues have enhanced reconstructive outcomes following tumor excision. These personalized therapeutic strategies not only help restore oral function and aesthetics but also provide valuable opportunities for studying oral cancer biology in a patient-specific manner [33]. In parallel, microfluidic chip models have revolutionized oral cancer research by accurately replicating the tumor microenvironment (TME). These chip-based systems enable real-time analysis of cellular interactions, tumor progression, and drug responses. Furthermore, they support high-throughput screening and mechanistic studies, thereby facilitating the identification of novel therapeutic agents and individualized treatment strategies tailored to specific patient profiles [33]. Oral cancer cell lines also play a vital role in advancing the understanding of oral carcinogenesis. These established in vitro models are widely used for investigating tumor biology, analyzing cellular mechanisms, and screening potential anticancer drugs. Characterization of oral cancer cell lines based on their genetic and phenotypic features provides important insights into tumor behavior, metastatic potential, and resistance to conventional therapies [34]. Recent developments in gene-editing technologies, particularly CRISPR/Cas systems, have further transformed oral cancer research. These powerful tools allow precise modification of genetic material, enabling targeted alterations in oncogenes and tumor suppressor genes. CRISPR-based approaches help researchers elucidate the functional role of specific genes in tumor initiation and progression while also identifying potential molecular targets for therapy [35]. In addition to genetic alterations, epigenetic modifications are critically involved in the development and progression of oral cancer. Environmental factors and lifestyle habits can induce reversible changes in gene expression through mechanisms such as DNA hyper methylation, histone modifications, and microRNA regulation. These epigenetic alterations influence oncogenes, tumor suppressor genes, apoptosis-related genes, and DNA repair pathways. Importantly, epigenetic changes are frequently associated with both early-stage lesions and advanced oral cancers, making them valuable biomarkers for diagnosis, prognosis, and therapeutic intervention. Consequently, epidrug therapy has emerged as a promising strategy aimed at reversing abnormal gene expression patterns and restoring normal cellular functions. Oral cancer remains a major public health concern in India, where it represents the most common cancer among males and the fifth most common cancer among females, contributing to nearly 26% of the global oral cancer burden. The major risk factors include tobacco consumption, alcohol use, and infection with high-risk Human Papillomavirus (HPV) types 16 and 18. However, only a small proportion of high-risk individuals with dysplastic lesions eventually develop oral cancer, indicating that individual genomic variations and epigenetic alterations play a crucial role in disease susceptibility and progression. Extensive studies have therefore focused on identifying molecular lesions and epigenetic biomarkers associated with oral carcinogenesis. The integration of autologous tissues, advanced chip models, oral cancer cell lines, gene-editing technologies, and epigenetic research represents a multidisciplinary framework for improving oral cancer management. Together, these innovative approaches offer significant potential for developing effective personalized therapies, enhancing tissue regeneration, and improving patient outcomes. Moreover, this comprehensive strategy contributes to a deeper understanding of the molecular mechanisms underlying oral cancer and establishes a strong foundation for future advancements in regenerative medicine and precision oncology [23]. By integrating biomaterials, cellular therapies, and bioactive factors, tissue engineering provides novel strategies for replacing or repairing damaged oral tissues. These approaches are increasingly influencing oral cancer treatment and reconstruction by promoting tissue regeneration, improving functional recovery, and supporting the development of personalized therapeutic interventions [36].

Scaffold-based approaches play a crucial role in tissue engineering for oral cancer treatment and reconstruction. In tissue engineering, scaffolds are three-dimensional structures designed to mimic the extracellular matrix (ECM) of natural tissues, thereby providing a supportive environment for cell attachment, proliferation, growth, and differentiation [37]. Various biomaterials, including hydrogels, collagen, gelatin, and bio ceramics, are widely utilized for scaffold fabrication in the regeneration of bone, soft tissues, and salivary glands affected by oral cancer [38]. Recent advancements in biomaterial engineering have led to the development of biodegradable scaffolds that gradually degrade as new tissue forms, eliminating the need for secondary surgical removal procedures [39]. In addition, these scaffolds can be incorporated with bioactive molecules such as growth factors and peptides to enhance tissue healing, angiogenesis, and vascularization, thereby improving regenerative outcomes [40].

### Bone regeneration

Bone regeneration is particularly important in oral cancer patients undergoing maxillary or mandibular resection, as restoration of bone structure is essential for functional and aesthetic rehabilitation [41]. Bioengineered scaffolds combined with mesenchymal stem cells (MSCs) have shown promising potential in promoting bone tissue regeneration and repair [42]. Furthermore, calcium phosphate-based biomaterials and three-dimensional (3D)-printed scaffolds have demonstrated significant effectiveness in stimulating new bone formation following tumor resection in maxillofacial surgery [43].

### Soft tissue reconstruction

**Fig. 2:**
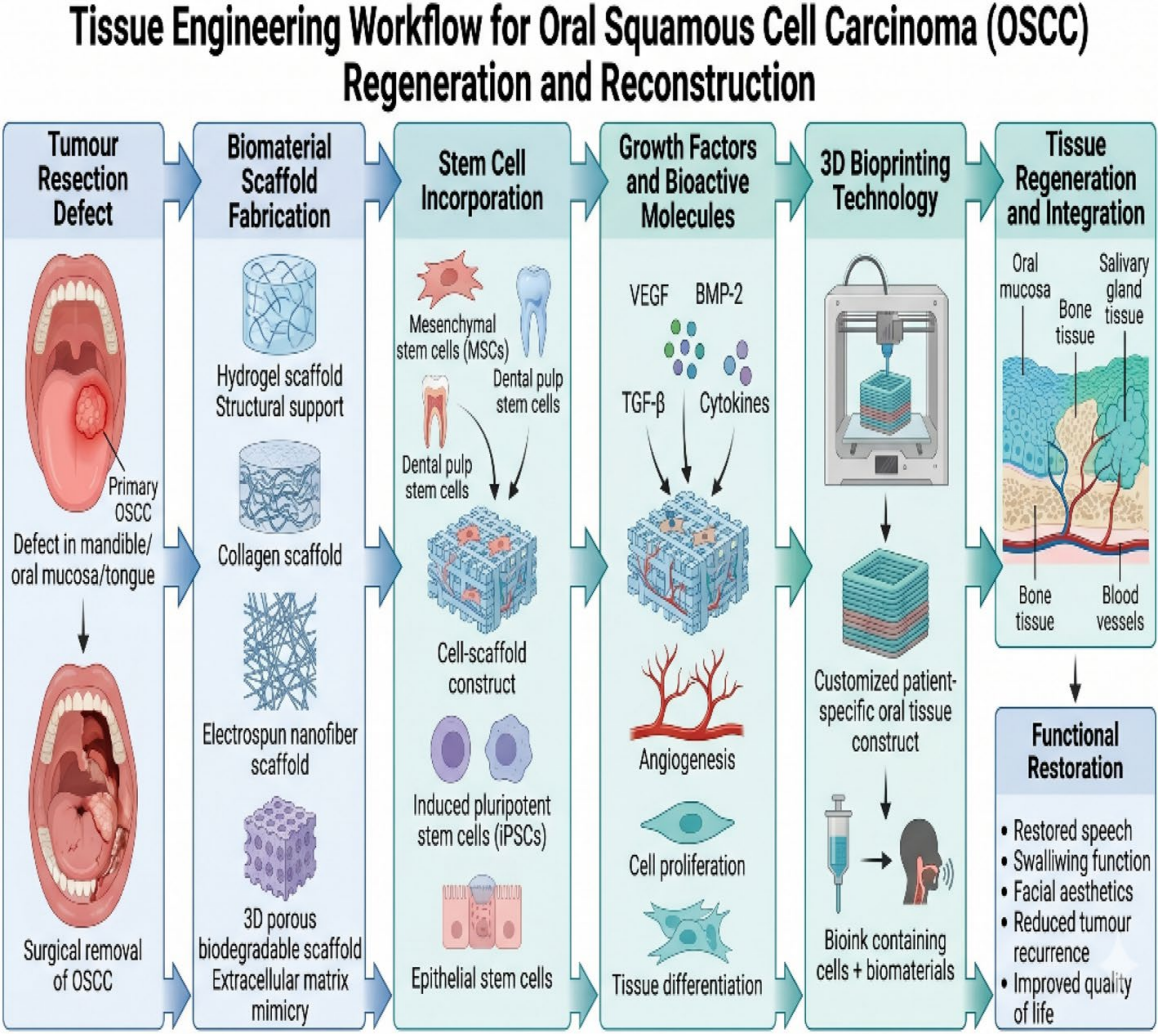
Tissue Engineering Workflow in OSCC

Soft tissue reconstruction is another critical aspect of oral cancer rehabilitation. Collagen- and gelatin-based scaffolds are commonly employed for the regeneration of oral mucosal tissues because they support epithelial cell attachment, proliferation, and tissue healing [44]. These scaffolds are particularly beneficial for repairing mucosal defects resulting from oral cancer surgery and associated treatments [45]. Additionally, electro spun fibrous scaffolds, which closely resemble the native fibrous architecture of oral soft tissues, are increasingly being incorporated into scaffold designs to improve tissue regeneration and functional integration [46].Tissue Engineering workflow for Oral Squamous Cell Carcinoma Regeneration and Reconstruction are summarised in Fig. 2.

### Cell-based therapies

Cell-based therapies represent one of the major focuses of tissue engineering in oral cancer treatment and reconstruction. Stem cells are of particular interest because of their remarkable ability to self-renew and differentiate into multiple specialized cell types, making them highly suitable for repairing and regenerating damaged tissues [47]. Various types of stem cells have been extensively investigated for their therapeutic potential in oral cancer management [48].

### Mesenchymal Stem Cells (MSCs)

Mesenchymal stem cells (MSCs) are multipotent stromal cells capable of differentiating into several tissue types, including bone, cartilage, and muscle cells [49]. MSCs derived from bone marrow, adipose tissue, and dental pulp have been widely utilized in tissue-engineering strategies for oral tissue regeneration [50]. In regenerative applications, MSCs are commonly seeded onto biocompatible scaffolds and stimulated to differentiate into specific target tissues, thereby replacing tissues lost or damaged following cancer treatment [37]. Their regenerative potential and immunomodulatory properties make MSCs particularly valuable in oral and maxillofacial reconstruction.

### Epithelial Stem Cells (ESCs)

Epithelial stem cells (ESCs) play a critical role in the regeneration and maintenance of the oral mucosa. These cells have the ability to differentiate into keratinocytes, which form the outer protective layer of the oral mucosal epithelium [51]. Tissue-engineered constructs containing ESCs are designed to accelerate wound healing, restore mucosal integrity, and improve oral functionality following surgery or radiation therapy [52]. Such approaches are especially important for repairing mucosal defects associated with oral cancer treatment.

### Induced Pluripotent Stem Cells (iPSCs)

Induced pluripotent stem cells (iPSCs) are generated by reprogramming adult somatic cells into an embryonic stem cell-like state, thereby enabling them to differentiate into virtually any cell type [53]. iPSCs offer significant potential for personalized regenerative medicine because patient-specific cells can be used to fabricate customized tissue-engineered constructs with minimal risk of immune rejection. Current research focuses on utilizing iPSCs for the regeneration of oral tissues damaged by cancer or its treatment.

Mesenchymal stem cells (MSCs) have emerged as highly promising regenerative candidates in oral squamous cell carcinoma (OSCC) owing to their immunomodulatory capacity, multilineage differentiation potential, and intrinsic tumor-homing ability [54]. Within the oral tumor microenvironment, MSCs regulate tissue repair and inflammatory responses through secretion of multiple paracrine mediators, including vascular endothelial growth factor (VEGF), platelet-derived growth factor (PDGF), fibroblast growth factor (FGF), transforming growth factor-β (TGF-β), and interleukin-10 (IL-10), which collectively contribute to angiogenesis, extracellular matrix remodeling, and wound healing [55]. MSC-derived extracellular vesicles and exosomes additionally mediate intercellular communication by transporting bioactive microRNAs, cytokines, and signaling molecules that influence cellular proliferation, immune modulation, and stromal remodeling within the tumor microenvironment [56]. Recent evidence indicates that MSCs exhibit dual and context-dependent functions in cancer biology. While MSCs may promote tissue regeneration and suppress excessive inflammation, they may also contribute to tumor progression through enhancement of epithelial-to-mesenchymal transition (EMT), angiogenesis, cancer stemness, and immune evasion under specific microenvironmental conditions [57]. Crosstalk between MSCs and tumor cells has been shown to regulate multiple oncogenic signaling pathways associated with metastasis and therapeutic resistance. Tumor-associated stromal interactions involving hypoxia-induced VEGF signaling, inflammatory cytokines, and extracellular matrix remodeling enzymes further facilitate angiogenic activation and tumor progression in OSCC [58]. Induced pluripotent stem cells (iPSCs) have also gained considerable attention in regenerative oral oncology because of their pluripotency and capacity for patient-specific tissue regeneration. IPSC-mediated regenerative mechanisms are primarily regulated through pluripotency-associated transcription factors such as OCT4, SOX2, and NANOG, which facilitate differentiation into osteogenic, epithelial, fibroblastic, and endothelial lineages relevant to oral and maxillofacial reconstruction [59]. Furthermore, iPSC-derived endothelial cells have demonstrated significant angiogenic potential and may contribute to vascular regeneration within engineered oral tissues. However, concerns regarding genomic instability, teratoma formation, and uncontrolled differentiation remain important translational limitations for clinical application [60]. The tumor microenvironment of OSCC is highly heterogeneous and consists of complex interactions among cancer stem cells (CSCs), immune cells, fibroblasts, endothelial cells, and inflammatory mediators [61]. CSC populations possess enhanced self-renewal capacity, tumor-initiating potential, and resistance to radiotherapy and chemotherapy, thereby contributing significantly to recurrence and metastatic dissemination. Emerging evidence suggests that exosome-mediated communication between CSCs and stromal cells regulates angiogenesis, immune suppression, and metastatic progression through multiple signaling pathways, including YAP1/HIF-1α, VEGF, and EMT-associated molecular networks [62]. Tumor-associated macrophages and immune infiltrates further influence angiogenic activation and tumor progression through cytokine-mediated signaling and exosomal microRNA transfer [63]. Moreover, distinct OSCC subtypes exhibit substantial molecular and immunological heterogeneity, resulting in variable therapeutic responses and regenerative outcomes [64]. Differences in angiogenic signaling, immune checkpoint activation, stemness-associated pathways, and stromal composition may significantly influence the efficacy of stem cell-based regenerative therapies [65]. Consequently, future regenerative strategies should incorporate patient-specific molecular profiling and precision-engineered biomaterial systems to optimize therapeutic efficacy and minimize tumor-promoting effects within the oral tumor microenvironment. Overview of Cell-Based Regenerative Strategies in Oral Squamous Cell Carcinoma are summarized in Table 1.

**Table 1:** Comparative Overview of Cell-Based Regenerative Strategies in Oral Squamous Cell Carcinoma

Cell Type	Mechanism of Action	Delivery Strategy	Regenerative/Therapeutic Effect	Limitations
Mesenchymal stem cells (MSCs)	VEGF, PDGF, IL-10 mediated angiogenesis and immunomodulation	Hydrogels, scaffold systems, injectable delivery	Tissue regeneration, anti-inflammatory effects	Potential tumour-promoting effects
Induced pluripotent stem cells (iPSCs)	OCT4/SOX2/NANOG-mediated pluripotency and differentiation	Biomaterial scaffolds, bio printed constructs	Bone and soft tissue regeneration	Genomic instability, teratoma formation
Oral stem cells	ECM remodelling and multilineage differentiation	Bioactive hydrogels	Periodontal and oral tissue regeneration	Limited clinical validation
Tumour-infiltrating lymphocytes (TILs)	Immune checkpoint regulation and cytotoxicity	Cell-mediated immunotherapy	Anti-tumour immune activation	Immune exhaustion
Cancer stem cells (CSCs)	EMT activation and self-renewal signalling	Targeted molecular therapy	Tumour progression and recurrence	Therapy resistance

### Salivary gland regeneration

Radiation therapy for oral cancer frequently damages salivary glands, leading to xerostomia (dry mouth) and associated complications that significantly affect patient quality of life. Tissue engineering has made considerable progress in salivary gland regeneration through the development of bioengineered glandular tissues and organoid systems [66]. Researchers are combining scaffold biomaterials with salivary gland stem/progenitor cells to promote the repair and regeneration of damaged glandular tissues [67].

Recent studies have demonstrated that hydrogels embedded with salivary gland cells can help restore saliva production in irradiated tissues [68]. In addition, tissue-engineered salivary gland organoids, which are miniature functional replicas of salivary glands, represent a promising advancement in regenerative therapy. These organoids may provide a functional replacement for damaged salivary glands in patients suffering from radiation-induced xerostomia [69].

### 3D bio printing in oral cancer reconstruction

Three-dimensional (3D) bio printing is one of the most advanced and innovative technologies in tissue engineering. This technique enables the precise fabrication of complex tissue structures through the layer-by-layer deposition of biomaterials, living cells, growth factors, and bioactive molecules collectively known as “bioinks” [70]. Because 3D bio printing allows the creation of patient-specific tissue constructs tailored to individual anatomical and functional requirements, it holds immense potential for personalized oral cancer reconstruction [71].

### Bone bio printing

3D bio printing has shown particular promise in mandibular reconstruction following tumor resection [72]. Researchers have successfully developed bio printed bone scaffolds loaded with osteogenic growth factors and stem cells to stimulate mandibular bone regeneration. These customized constructs can be precisely designed to fit patient-specific defects, thereby improving structural integration, accelerating healing, and enhancing functional outcomes [73].

### Soft tissue bio printing

In addition to bone regeneration, 3D bio printing is increasingly being applied for the fabrication of oral soft tissues, including mucosal and muscular tissues required for oral reconstruction [74]. Customized bio printed soft tissue constructs may significantly improve rehabilitation outcomes in patients undergoing treatment for oral cancer by restoring tissue function, aesthetics, and overall quality of life [75].

### Autologous tissues in oral cancer treatment

Autologous tissue plays a vital role in the treatment and reconstruction of patients undergoing surgery for oral cancer. Following tumour excision, significant functional and aesthetic defects often occur within the oral cavity and surrounding structures. The use of autologous tissue helps restore both form and function, thereby improving speech, swallowing, appearance, and overall quality of life in affected patients [76].

### Role of autologous tissue in oral cancer treatment

#### Reconstruction after tumour resection

Surgical resection of oral tumours frequently results in extensive soft tissue and bone defects that require reconstruction. Autologous tissue is commonly utilized for restoring these defects because of its excellent compatibility and regenerative capacity [77].

#### Soft tissue reconstruction

Autologous flaps are widely used to repair soft tissue defects created after removal of malignant tissues. Among the most commonly employed flaps are the radial forearm free flap (RFFF) and fibula free flap. The radial forearm free flap is highly versatile and provides both skin and mucosal tissue, making it suitable for reconstruction of oral cavity and oropharyngeal defects while restoring oral functionality. The fibula free flap is extensively used for mandibular reconstruction because it provides vascularized bone along with soft tissue support, thereby facilitating both functional recovery and satisfactory cosmetic outcomes.

#### Bone reconstruction

Autologous bone grafts are particularly important for reconstruction of mandibular defects following tumour resection. Among available options, the fibula free flap remains one of the most preferred techniques because it offers vascularized bone tissue that promotes improved healing, structural stability, and long-term functional rehabilitation [78].

#### Functional reconstruction

The primary objective of reconstructive surgery in oral cancer patients is to restore essential oral functions such as speech, swallowing, and salivary secretion.

#### Speech and swallowing restoration

Following procedures such as partial or total glossectomy, reconstructive surgery using autologous muscle or skin flaps obtained from the forearm or thigh can help recreate a functional tongue structure. This significantly improves speech articulation and swallowing ability in affected patients [79].

#### Salivary gland reconstruction

In certain cases, salivary glands damaged or removed during oral cancer surgery require reconstruction. Autologous tissue can be utilized to rebuild or reposition glandular tissues, thereby helping restore saliva production and reducing complications such as xerostomia [80].

#### Advantages of autologous tissue

Autologous tissue reconstruction offers several significant advantages in oral cancer treatment:

##### Reduced risk of rejection

Since the tissue is derived from the same patient, the likelihood of immunological rejection is minimal.

##### Faster tissue integration

Autologous grafts integrate more effectively with recipient tissues, resulting in superior functional and aesthetic outcomes [81].

##### Lower risk of infection

Compared with allogeneic grafts, autologous tissues generally exhibit a lower incidence of postoperative infection, making them highly suitable for oral reconstruction [82].

#### Challenges associated with autologous tissue reconstruction

Despite its benefits, autologous tissue reconstruction also presents certain challenges. Harvesting tissue from donor sites may lead to donor site morbidity, including pain, scarring, and functional limitations [83]. Additionally, autologous tissue harvesting and transfer require technically demanding surgical procedures, increasing the complexity and risk of operative complications [84].

#### Advances in autologous tissue engineering

Recent advancements in tissue engineering have significantly improved the success of autologous tissue reconstruction. Techniques involving stem cell therapy, vascularized tissue grafts, and 3D bio printing have enhanced tissue regeneration and graft survival. Researchers are particularly focusing on improving vascularization within tissue flaps to minimize graft failure and improve long-term outcomes [85].

Overall, autologous tissue remains a cornerstone in oral cancer reconstruction because it offers improved functional recovery, enhanced aesthetics, lower infection rates, and reduced immunological complications. Ongoing developments in regenerative medicine and tissue engineering continue to optimize these reconstructive strategies and may further improve patient outcomes in the future [86].

### Mechanism of autologous tissue in oral cancer treatment

#### Tissue regeneration and integration

Autologous tissue promotes efficient tissue regeneration and integration because it is biologically compatible with the recipient site. Following transplantation, several biological mechanisms contribute to successful healing and tissue restoration.

#### Cell migration and proliferation

Cells present within transplanted tissues, including skin, muscle, and bone cells, migrate into the wound area where they proliferate and initiate the formation of new tissue. This process is essential for covering tissue defects and restoring normal tissue architecture [87].

#### Angiogenesis

The formation of new blood vessels, known as angiogenesis, is critical for graft survival. Adequate vascularization ensures continuous oxygen and nutrient supply to the transplanted tissue, thereby supporting successful integration and healing [88].

#### Extracellular matrix formation

Autologous grafts facilitate the deposition of a new extracellular matrix (ECM), which serves as a structural scaffold for tissue remodelling and collagen production. The ECM is essential for maintaining graft stability and promoting long-term tissue regeneration [89].

#### Vascularization

Successful transplantation of vascularized flaps, such as the fibula free flap or radial forearm free flap, depends on surgical anastomosis of donor blood vessels with recipient-site vessels. This process supports:

##### Immediate blood flow

Rapid restoration of circulation prevents tissue ischemia and promotes early healing.

##### Sustained graft survival

Progressive angiogenesis from recipient tissues improves long-term graft viability and integration [90].

#### Restoration of function

Restoration of oral functionality is a primary objective of autologous tissue reconstruction.

#### Functional muscle and tissue integration

When muscle or skin flaps are used to reconstruct structures such as the tongue, palate, or facial tissues, the integrated tissues can gradually regain function, thereby improving speech and swallowing capabilities.

#### Bone regeneration

Autologous bone grafts, particularly fibular grafts, restore structural integrity in patients undergoing mandibular resection. This contributes to facial symmetry, dental occlusion, and overall oral function [91].

#### Salivary gland function restoration

Autologous reconstruction of salivary gland tissues may help restore salivary secretion, reduce oral dryness, and improve quality of life following glandular damage during oral cancer surgery [81].

#### Reduced risk of rejection

Because autologous tissues originate from the patient’s own body, the immune system recognizes them as “self,” thereby minimizing immune-mediated graft rejection. This leads to improved graft survival, reduced need for immunosuppressive therapy, and faster healing processes [92, 93].

#### Scar formation and aesthetic restoration

An additional advantage of autologous tissue reconstruction is the achievement of superior aesthetic outcomes. Skin or mucosal grafts harvested from donor sites such as the forearm or thigh can closely match the texture and appearance of oral tissues, thereby improving facial aesthetics and psychological well-being following surgery [94].

**Fig. 3:**
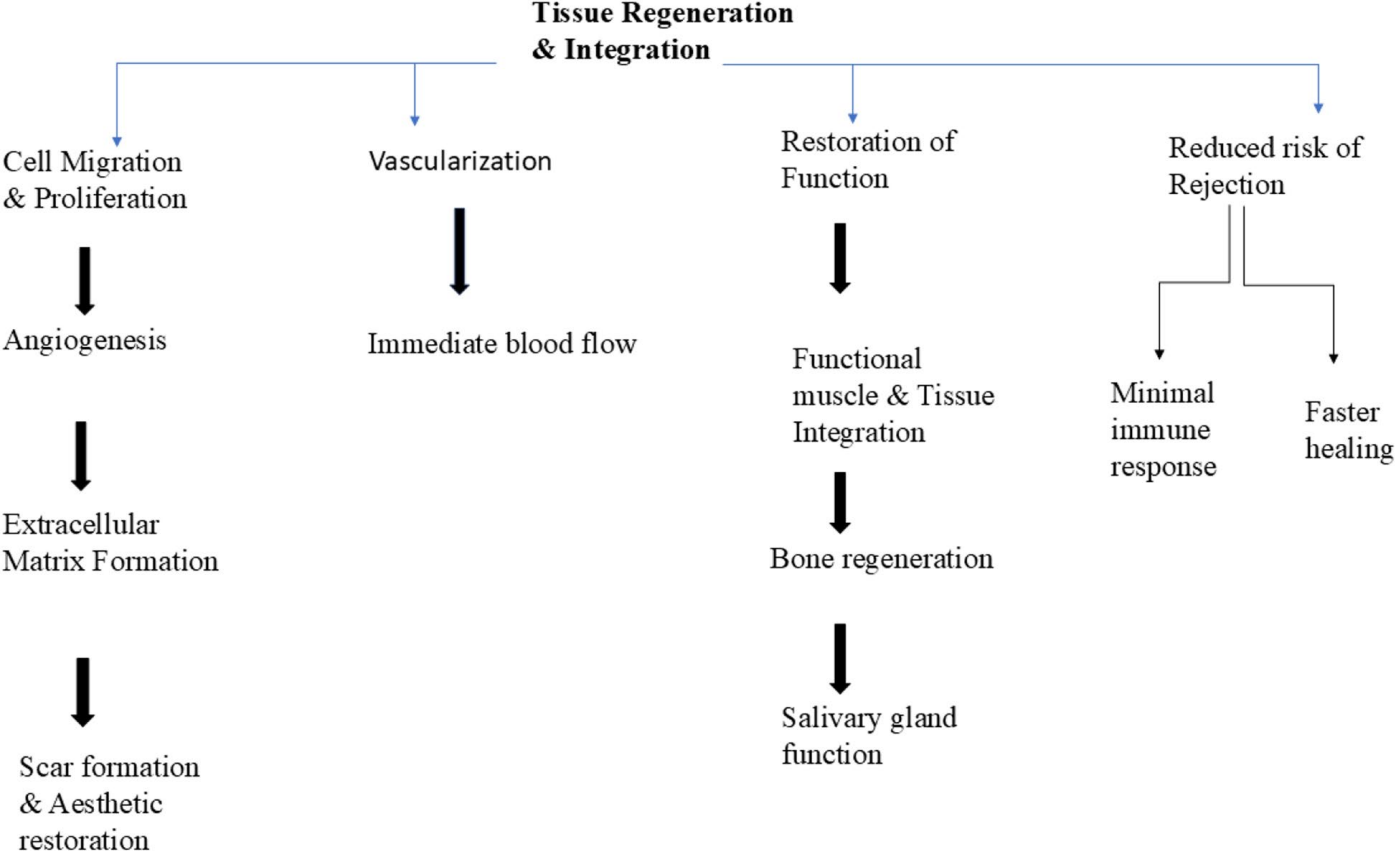
Mechanism of Autologous Tissue in Oral Cancer Treatment

In summary, autologous tissue reconstruction in oral cancer treatment relies on natural biological mechanisms including tissue regeneration, vascularization, extracellular matrix formation, and functional integration. These processes collectively promote healing, restore oral function, minimize graft rejection, and improve both functional and cosmetic recovery. Ongoing advances in tissue engineering and stem cell research are expected to further enhance the effectiveness and long-term success of autologous tissue-based therapies in oral cancer management [95]. Mechanism of Autologous Tissue in Oral Cancer Treatment are summarised (Fig. 3).

## Chip model – repertoire of biological model system

Biomedical research has undergone a revolution with the advent of organ-on-a-chip technologies, especially in the field of cancer studies. Chip models are proving to be very useful research instruments for oral cancer because they mimic the physiological conditions of human tissues [86]. In the context of treating oral cancer, the CHIP model—which stands for "Cellular Heterogeneity, Immune Profiling, and Phenotypic Repertoire"—is a paradigm that effectively encapsulates the intricacies of cancer biology and therapeutic responses. Through the integration of several biological systems and processes, this model helps to better understand immunological dynamics, phenotypic plasticity, tumour heterogeneity, and how these aspects affect treatment resistance and efficacy in cancers such as oral squamous cell carcinoma (OSCC) [13]. The CHIP model contributes to a comprehensive understanding of oral cancer treatment by considering the interactions between cancer cells and the immune system, the ways in which their phenotypic traits change in response to treatment, and the ways in which they display distinct behaviours depending on environmental and genetic factors [96].

### CHIP model in oral cancer treatment: a repertoire of biological model systems

Three crucial elements are highlighted by the CHIP model: Three crucial elements are highlighted by the CHIP model:

1. Cellular Heterogeneity
2. Immune Profiling
3. Phenotypic Repertoire [97]

#### Cellular heterogeneity in oral cancer treatment

Oral malignancies, especially oral squamous cell carcinoma (OSCC), are characterized by their genetic heterogeneity, which means that several cell subpopulations with unique molecular traits make up the tumour. Because distinct tumour cell types may react differently to chemotherapy, radiotherapy, or immunotherapy, this heterogeneity makes treatment more difficult [10].

##### Tumour evolution

Cancer cells may experience epigenetic modifications and genetic alterations that enable them to adjust to various environmental stressors, including immunological surveillance or treatment. For instance, OSCC frequently has changes in cell cycle regulators, EGFR overexpression, and p53 mutations [11].

##### Cancer Stem Cells (CSCs)

Cancer stem cells are a subset of cells that may be responsible for metastasis, tumour growth, and treatment resistance. Even after tumours appear to be destroyed, these CSCs can rebuild them. In the CHIP model, they are a crucial target because they frequently exhibit greater resistance to radiation and chemotherapy [12].

##### Heterogeneous Tumour Microenvironment (TME)

**Fig. 4:**
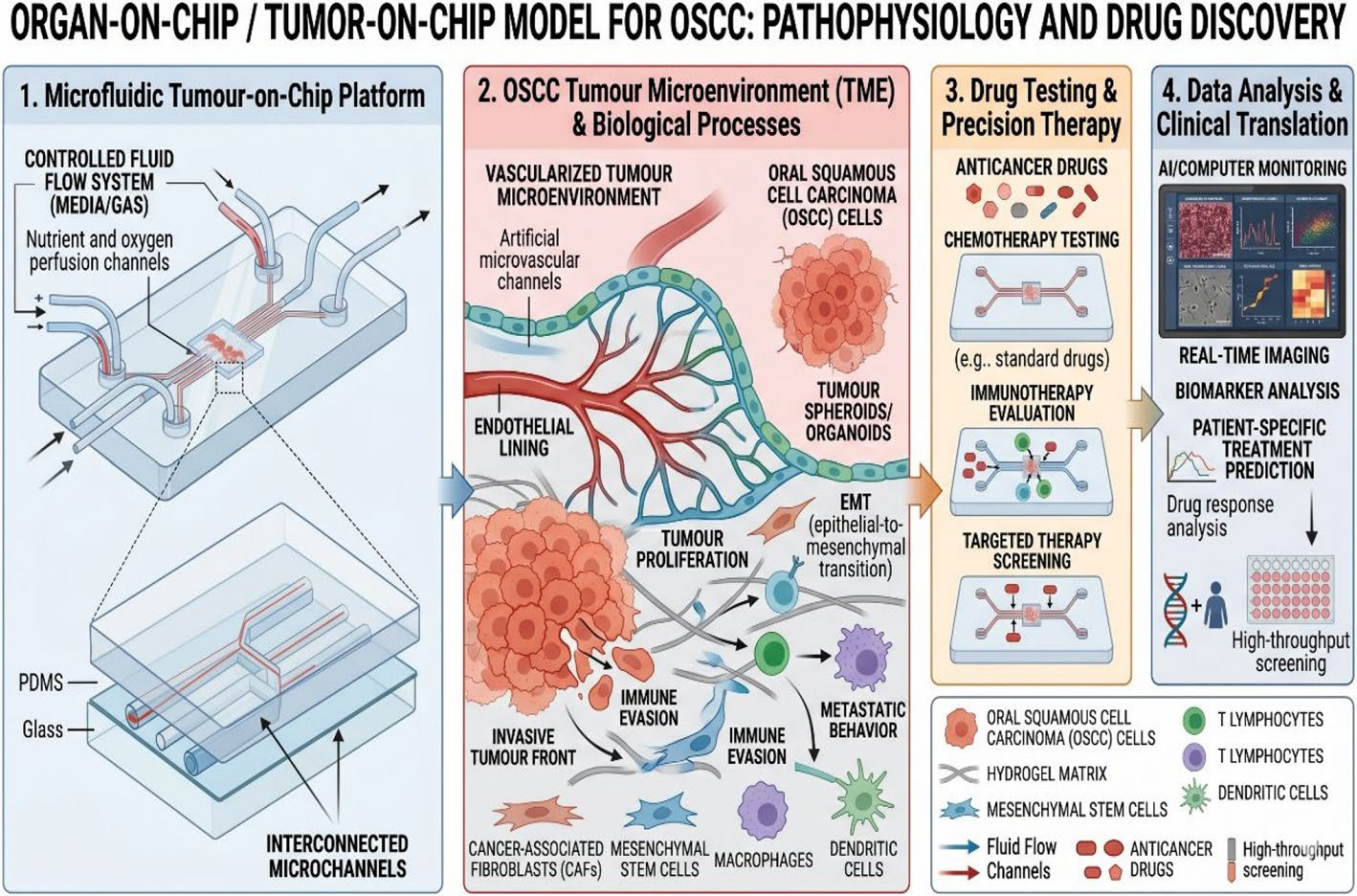
Organ-on-Chip

Fibroblasts, endothelial cells, cancer cells, and other immune cells comprise the TME. The way these cells interact can either suppress immune responses or encourage the growth of tumours, which can affect how well treatments work. The CHIP model examines how cellular heterogeneity and treatment resistance are influenced by this environment [13]. Tumour on chip model on Oral Squamous Cell Carcinoma are summarized in Fig. 4.

#### Example in OSCC

Clonal selection may be used to generate tumours in oral squamous cell carcinoma, with some clones becoming dominant due to genetic advantages such as resistance to apoptosis (e.g., lack of p53 function). Improving the targeting of treatments, such as immunotherapies or targeted therapies, requires an understanding of the clonal evolution within malignancies [98].

##### Immune profiling in oral cancer treatment

Understanding how oral malignancies elude the immune system and how treatments can take advantage of or strengthen immune responses depends on immunological profiling. According to the CHIP model, immune profiling entails determining the types of immune cells found in the TME and their functional status in addition to comprehending the ways in which cancers elude immune monitoring [99].

##### Immune escape mechanisms

Immune checkpoint inhibitors, such PD-L1, can be used by oral malignancies to elude immune responses. These malignancies might increase the production of PD-L1, which attaches to T-cell PD-1 receptors to prevent T-cell activation and enable the tumour to continue growing. The use of checkpoint inhibitors, such as pembrolizumab and nivolumab, to re-activate immune responses is incorporated into the CHIP model [100].

##### Immunosuppressive TME

Immunosuppressive cells such tumour-associated macrophages (TAMs), myeloid-derived suppressor cells (MDSCs), and regulatory T cells (Tregs) are frequently found in the TME. Tumour development may result from these cells' inhibition of T-cell activity. The CHIP model explores the interactions between these cells and tumour cells as well as the ways in which treatments can target this immunosuppressive milieu [101].

##### Tumour-Associated Antigens (TAAs)

Finding certain TAAs in oral cancer can aid in the creation of focused immunotherapies. In oral malignancies that are HPV-positive, for instance, tumour-specific human papillomavirus (HPV)-associated antigens have been found, and vaccines that target these antigens are being investigated [102].

#### Example in OSCC

Tumour-infiltrating lymphocytes (TILs) may be more prevalent in HPV-positive OSCC, and immunological profile can forecast the propensity for immune checkpoint inhibitor response. However, the microenvironment of HPV-negative OSCC is typically more immunosuppressive, necessitating the use of alternative therapeutic approaches, such as combination immunotherapy [45].

##### Phenotypic repertoire in oral cancer treatment

The ability of cancer cells to alter their phenotype in response to external stimuli, such as immunological pressure or treatment, is known as phenotypic plasticity. This has a significant impact on how oral malignancies change throughout the course of treatment and is a critical element in tumour growth and drug resistance [14].

##### Epithelial-to-Mesenchymal Transition (EMT)

Through the process of epithelial-mesenchymal transition (EMT), tumour invasion and metastasis are encouraged. EMT plays a significant role in the development of oral cancer, especially in the capacity of cancer cells to move to other locations. EMT is a crucial factor for targeted therapy since the CHIP model considers how it affects immunoresistance and chemo resistance [15].

##### Plasticity in drug response

By changing their phenotypic, oral cancer cells can become resistant to radiation or chemotherapy. For example, certain cells may engage DNA repair systems to fend off radiation-induced damage, while others may upregulate ABC transporters that release chemotherapeutic medicines. The CHIP model forecasts the potential impact of phenotypic changes on treatment outcomes and assists in identifying biomarkers of drug resistance [16].

#### Example in OSCC

The capacity of tumour cells to undergo EMT in OSCC can result in metastasis and invasive behaviour, making treatment more difficult. By evaluating the correlation between EMT indicators (such as vimentin and E-cadherin) and the response to radiation or chemotherapy, the CHIP model facilitates the creation of more individualized and efficient treatment plans [103].

#### Technologies supporting the CHIP model in oral cancer treatment

The CHIP model is supported by several innovative technologies that aid in the better understanding and management of oral malignancies by researchers and clinicians [104].

##### Organoids and 3D tumour models

3D models made from patient tumours that preserve important aspects of the original tissue are called organoid cultures. Compared to conventional 2D cultures, these models provide a more physiologically appropriate setting for studying tumour heterogeneity and treatment responses [105].

##### Single-Cell RNA Sequencing (scRNA-seq)

By identifying gene expression at the single-cell level, single-cell RNA sequencing offers comprehensive insights into the diversity of immune and tumour cells inside the TME. This can direct the creation of more focused treatments for OSCC [106].

##### Tumour-on-a-chip devices

Real-time tumour behaviour monitoring and therapy response testing are made possible by the development of miniature, dynamic settings that mimic the TME using microfluidic tumour-on-a-chip models. These models can be used to predict medication resistance and assess the efficacy of immunotherapy and chemotherapy [107].

The CHIP model, which combines cellular heterogeneity, immunological profiling, and phenotypic plasticity to offer a thorough grasp of tumour biology and therapeutic obstacles, is a useful and developing method for studying and treating oral cancer. This model can help researchers and doctors create more individualized treatment plans, combat drug resistance, and enhance patient outcomes [108].

## Oral cancer cell line

Oral cancer treatment and cancer research both benefit greatly from the use of oral cancer cell lines. With the use of these cell lines, scientists can investigate the biology of oral malignancies, assess therapeutic responses, and create novel therapies. The most prevalent kind of oral cancer is oral squamous cell carcinoma (OSCC), and several OSCC cell lines have been developed to study different facets of tumour biology, including cellular mechanisms, drug resistance, and immune evasion [109].

An outline of oral cancer cell lines function in oral cancer treatment, including mechanisms and their usage in testing novel therapies, is provided here.

### Role of oral cancer cell lines in oral cancer treatment

Tumour Biology and Mechanisms of Disease: To comprehend the molecular mechanisms behind tumour formation, metastasis, and treatment resistance, oral cancer cell lines—especially those obtained from oral squamous cell carcinoma (OSCC)—are crucial. Key signalling pathways linked to epithelial-to-mesenchymal transition (EMT), cell-cycle regulation, and oncogene activation (e.g., EGFR, PIK3CA) can be investigated by researchers [110].

Chemotherapy and Radiation: Oral cancer cell lines offer platforms for assessing the sensitivity to radiation and chemotherapy. To find effective treatment plans and learn how cancer cells react to various chemotherapeutic agents (such as cisplatin, 5-fluorouracil, and paclitaxel) and radiation at the cellular level, researchers test these treatments on cell lines [111].

Because oral malignancies, particularly OSCC, frequently become resistant to common therapies including chemotherapy and radiation, drug resistance studies are essential. Cell lines are useful in determining the mechanisms underlying this resistance, such as drug efflux pump upregulation, apoptosis pathway alteration, or DNA repair processes [112].

Targeted Therapy: Based on molecular changes, a variety of OSCC cell lines are employed to find targeted therapies. For instance, EGFR inhibitors like as cetuximab or erlotinib can target the overexpression of epidermal growth factor receptor (EGFR) in OSCC. The effectiveness of such targeted therapies, which may offer a more accurate method than traditional chemotherapy, is assessed with the use of oral cancer cell lines [113].

Immunotherapy: The efficacy of immunotherapy, such as immune checkpoint inhibitors (e.g., anti-PD-1 and anti-PD-L1 treatments), CAR-T cell therapies, and vaccines, is also investigated using oral cancer cell lines. To assess how the tumour microenvironment (TME) affects immune responses, these cell lines can be modified to express pertinent antigens or immunological checkpoints. These cell lines can be immunoprofiled to find possible indicators for clinical trial patient selection. In OSCC cell lines, for instance, PD-L1 expression is investigated to determine whether it may be related to immune evasion and the reaction to immune checkpoint inhibitors [114].

Preclinical Drug Discovery and High-Throughput Screening: High-throughput screening (HTS) employs oral cancer cell lines to examine vast pharmacological libraries and find possible clinical trial candidates. This method aids in the discovery of new substances or drug combinations that may be more useful in the treatment of OSCC [115].

Furthermore, because they more closely resemble the tumour microenvironment (TME) and tumour-stroma interactions, which are essential for forecasting therapeutic responses and researching drug resistance," organotypic cultures and 3D models made from oral cancer cell lines are becoming more and more popular [116].

### Common oral cancer cell lines used in research and treatment

Here are some commonly used oral cancer cell lines, many of which are derived from OSCC, and their relevance to oral cancer treatment:

#### HSC-3

Origin: Human oral squamous cell carcinoma from the tongue.

Characteristics: Extremely aggressive, metastatic, and frequently used to investigate oral cancer's invasion and metastasis.

Applications: The evaluation of chemo resistance and radiotherapy resistance to cisplatin and other chemotherapy drugs is done using HSC-3 cells [117].

#### Cal-27

Origin: Human oral squamous cell carcinoma derived from the tongue.

Characteristics: utilized in research projects looking at radiation resistance and chemo resistance. Investigating molecular therapies that target EGFR is made easier with this cell line.

Applications: Research on EGFR-targeted therapy frequently uses Cal-27 cells to examine the part oncogene addiction plays in OSCC [118].

#### SCC-9

Origin: Human OSCC from the tongue.

Characteristics: Sensitive to chemotherapy drugs, including cisplatin.

Applications: Studies on chemo response and drug resistance benefit from the use of SCC-9 cells, particularly when assessing novel chemotherapy combinations or targeted therapies [119].

#### FaDu

Origin: Human head and neck squamous cell carcinoma (HNSCC) from the hypopharynx.

Characteristics: FaDu cells are frequently employed to investigate cell signalling pathways and radiation resistance that contribute to the development of tumours.

Applications: Because head and neck tumours are comparable to oral cancer, FaDu cells can be used for radiotherapy and chemotherapy combination studies as well as for examining the mechanisms of metastatic spread [120].

#### UM-SCC-1 and UM-SCC-22A

Origin: Human oral squamous cell carcinoma (OSCC).

Characteristics: These cells, which are derived from oral cavity cancers, have been extensively utilized to investigate immune checkpoint inhibition and EGFR-targeted therapies.

Applications: These cell lines are used to assess the effectiveness of combination treatments and immune checkpoint inhibitors.

#### Tca8113

Origin: Human OSCC derived from gingival tissue.

Characteristics: Research on drug metabolism and chemo resistance is conducted using this cell line.

Applications: Tca8113 cells have been utilized to assess combination therapies for oral cancer and investigate paclitaxel resistance [121].

Mechanisms of Oral Cancer Cell Lines in Treatment Studies:

Researchers can examine several important therapy and resistance mechanisms using oral cancer cell lines, including.

Drug Resistance Mechanisms: A lot of oral cancer cell lines eventually become resistant to common treatments. The causes of this resistance may include upregulation of ABC transporters (which carry medications out of cells), DNA repair processes, or changes in apoptosis signalling (e.g., loss of p53 activity) [122].

Tumour Microenvironment (TME) and Drug Response: One important factor affecting treatment results is the TME. Cells in the tumour microenvironment (TME), such as tumour-associated macrophages (TAMs) and cancer-associated fibroblasts (CAFs), can release cytokines that aid in the tumour’s immune evasion or encourage resistance to treatment. To investigate these relationships and test medications that can alter the TME, oral cancer cell lines are utilized [123].

Immunotherapy and Immune Evasion: To evaluate the effectiveness of immune checkpoint inhibitors, which prevent PD-1/PD-L1 from interacting and help the immune system identify and eliminate tumour cells, a variety of OSCC cell lines are employed. How these cancers evade immune monitoring is better understood by using the CHIP model, which looks at immune profile in oral cancer cell lines [124].

Metastasis and Invasion: The mechanisms of tumour invasion and metastasis are frequently studied using cell lines like Cal-27 and HSC-3. Developing therapies that stop oral cancer from spreading to other body areas requires an understanding of these pathways [125].

The development of new treatments for oral malignancies, particularly oral squamous cell carcinoma (OSCC), depends on oral cancer cell lines. They shed light on drug resistance, immune evasion, and the biological mechanisms of cancer progression. Researchers can use these cell lines to screen for medicines such as immunotherapies, targeted therapies, radiotherapy, and chemotherapy that work. They are therefore essential in enhancing the personalization of oral cancer therapies and resolving issues related to metastasis and resistance [126].

### CRISPR and gene editing technologies

With new ways to prevent cancer recurrence in patients with oral cancer, CRISPR-Cas9 gene-editing technology is transforming the field of tissue engineering. Two major obstacles in the treatment of oral cancer are repairing damaged tissue and stopping the spread of cancer cells. CRISPR's precision enables researchers to target genes that are involved in both tumour suppression and tissue regeneration. With the use of this technology, doctors can now focus on the genetic alterations that promote the spread of cancer. This presents a highly focused therapeutic option that lessens the side effects of conventional cancer treatments like radiation and chemotherapy [127]. In tissue engineering, improving the engineered tissues' capacity to suppress tumours is one of the main areas of interest for CRISPR. Mutations in the DNA that enable oral cancer cells to avoid the body's defences and grow and spread aggressively are frequently seen in these cells. Scientists can rewire cells in the engineered tissues to prevent the growth of tumours by altering these genes. In cases of oral cancer, this may lessen the likelihood of local recurrence following surgical resection. The application of CRISPR limits the potential of cancer to spread to other parts of the body by specifically targeting tumour suppressor genes or oncogenes [105].

Beyond its use in tumour suppression, CRISPR is being used to encourage quicker and more effective tissue healing following oral cancer surgeries. Speech and eating abilities can be severely hampered by tissue loss from radiation therapy or tumour removal, which can also seriously lower a patient's quality of life [14]. To improve the body's innate healing process, scientists are looking into ways to modify the genes that control cell division, proliferation, and tissue regeneration. CRISPR has the potential to enhance the functional and aesthetic restoration of oral tissues by hastening the regeneration of new tissue in patients undergoing reconstructive surgeries [107].

CRISPR-Cas9's potential for personalized medicine holds the key to its future in the treatment of oral cancer. With a better grasp of the genetic alterations unique to each patient's tumour, researchers will be able to create more specialized gene-editing plans that are catered to the requirements of each patient. With its highly effective and minimally invasive therapeutic options, this degree of customization has the potential to transform not only the treatment of oral cancer but also the larger field of tissue engineering [15]. Most of this research is still in the experimental stage, but the initial findings are encouraging and suggest that CRISPR may eventually be used routinely for tissue repair as well as cancer suppression. Mutations in important genes, especially oncogenes and tumour suppressor genes, are frequently observed in oral cancer. By selectively editing these genes, researchers can examine how they affect the development of cancer using CRISPR-Cas9. As an illustration, the TP53 gene, sometimes known as the "guardian of the genome," is essential for controlling both apoptosis and cell division. A significant portion of oral cancers have TP53 mutations. To investigate how these mutations affect tumour initiation and progression, lab models can be engineered with CRISPR. Likewise, to learn more about the function of tumour suppressor genes like NOTCH1 in the etiology of oral cancer, scientists are employing CRISPR to eliminate these genes [106].

Gene therapies for oral cancer are another area of research into CRISPR's potential. Several oral cancers are caused by genetic mutations that promote cancerous growth. The more potent treatments are raised by CRISPR's capacity to fix these mutations. Two proteins that control the progression of the cell cycle are encoded by the gene CDKN2A: p16 and p14. With oral squamous cell carcinoma, CDKN2A inactivation is frequently seen [16]. To restore normal cell cycle regulation and possibly stop the spread of cancer, researchers are employing CRISPR-Cas9 to fix mutations in CDKN2A. Offering a more individualized approach to cancer care, such precision-based therapies may be used in addition to current treatments such as radiation and chemotherapy [109].

In addition to gene therapy, CRISPR plays a critical role in creating animal models of oral cancer that closely resemble human cases. The intricacy of human tumours is frequently not accurately simulated by conventional cancer models, which makes it challenging to successfully test novel treatments. Using CRISPR, researchers can modify human oral cancers by introducing mutations into mouse models. These genetically modified models shed light on the progression and therapeutic response of oral cancer. For example, scientists can produce animal models that closely resemble human disease by editing genes such as EGFR or PIK3CA, both of which are frequently mutated in oral cancer. These models serve as a testing ground for experimental therapies and aid in the expeditious development of new treatments [110].

Although genetic mutations are a major cause of oral cancer, heritable modifications known as epigenetics—which modify gene expression without changing the DNA sequence—also have a substantial impact. CRISPR-Cas9 has been modified to investigate these modifications. Scientists can modify gene expression without altering the genome by employing a CRISPR variation known as CRISPR interference (CRISP RI). This enables scientists to investigate how epigenetic changes, like DNA methylation or histone modifications, impact the onset of oral cancer. For instance, aberrant methylation patterns in tumour suppressor genes can mute the expression of those genes, advancing the development of cancer. Through the ability to undo these epigenetic modifications and investigate their consequences, CRISP RI may provide access to novel therapeutic options [111].

Drug resistance development is one of the main treatment challenges for oral cancer. Chemotherapy and other treatments often cause many patients to respond well at first, but tumour resistance frequently develops, which can result in relapse. The genetic pathways underlying medication resistance in oral cancer are being investigated through CRISPR. Researchers can determine which genetic alterations allow cancer cells to withstand treatment by methodically knocking out genes thought to be involved in resistance. Developing strategies to overcome resistance requires knowledge of this kind. For instance, using CRISPR, researchers have found genes linked to resistance to cisplatin, a common chemotherapy medication used to treat oral cancer. By focusing on these genes, current therapies may become more efficacious [112].

Like numerous other cancers, oral cancer is skilled at dodging the body's defences. The presence of blood vessels, fibroblasts, and immune cells in the tumour microenvironment is essential for cancer cells to avoid immune recognition. Oral cancer cells and their surrounding microenvironment are interacting, and this is being studied using CRISPR. To prevent immune attack, cancers use genes like PD-L1, which are involved in immune checkpoint pathways, to be knocked out by researchers using CRISPR. Gaining knowledge about how oral cancer affects the immune system may help develop immunotherapies that strengthen the body's defences against the illness [103].

Patient outcomes are greatly enhanced by early detection of oral cancer; however, trustworthy biomarkers are still unobtainable. New genetic markers for early-stage oral cancer are being identified and validated using CRISPR technology. Researchers can determine which genetic changes are linked to the onset of the disease by editing genes suspected of being involved in the early development of cancer. The development of diagnostic tools that identify oral cancer in its early stages, when it is most curable, may result from these findings. Notch1 and FAT1 gene mutations are examples of possible biomarkers that could be used for early detection in high-risk populations and are already being found by CRISPR-based research [113].

Enhancing the response of oral cancer to conventional treatments such as chemotherapy and radiation therapy is another intriguing use of CRISPR. CRISPR can increase cancer cells' sensitivity to these treatments by deleting particular genes. For instance, CRISPR is being used by researchers to stop oral cancer cells' DNA repair mechanisms. Disabling the cancer cells' capacity to repair their DNA may increase their susceptibility to treatments like radiation and many chemotherapies, as these methods cause damage to DNA. Preclinical research on this approach is already demonstrating promise, and it may result in combination treatments that are more successful in eradicating cancer cells [114].

CRISPR-Cas9 can be used for "gene knock-in" techniques, in which scientists introduce functional copies of genes into cells, in addition to gene knockouts. This method works especially well for activating tumour suppressor genes that are deactivated in cases of oral cancer [115] For instance, introducing a functional p53 gene knock in may aid in re-establishing regular cell cycle regulation and halting the development of cancer. Although gene knock-in techniques are still in the experimental phase, they have enormous potential for creating targeted treatments that directly target the genetic flaws causing oral cancer [128].

### Epigenetic modifications

As an additional strategy to direct genetic engineering methods, epigenetic modifications have become a major focus in the research and treatment of oral cancer. Epigenetic modifications are heritable adjustments in gene expression that do not affect the DNA code itself, in contrast to genetic mutations that change the DNA sequence. These alterations have a major Impact on the development of cancer and are essential in the control of gene activity. By inhibiting tumour suppressor genes or turning oncogenes, aberrant epigenetic changes like DNA methylation and histone modification aid in the development of oral cancer. To undo the epigenetic alterations that encourage the development of cancer, researchers are looking into these modifications as possible therapeutic targets. It explores the different t pathways that contribute to oral cancer and how epigenetic treatments may enhance the effectiveness of treatment [17].

One of the most well-studied epigenetic changes is called DNA methylation, and it occurs when a methyl group is added to the cytosine base of DNA, especially in CpG islands, which are areas of the genome that are high in guanine and cytosine nucleotides. Methylation aids in the regulation of gene expression in healthy cells, keeping some genes dormant when not required [18].

Aberrant DNA methylation patterns are frequently found in cases of oral cancer, nevertheless. Tumour suppressor genes, like MLH1 and CDKN2A, are frequently hyper methylated, which silences them. Because of this silencing, vital regulatory controls on cell growth are eliminated, allowing malignant cells to multiply unchecked. Through comprehending the distinct methylation patterns associated with oral cancer, scientists aim to create treatments capable of undoing these modifications [19].

DNA methyl transferase (DNMT) inhibitors are a promising strategy to treat aberrant DNA methylation in oral cancer. Enzymes known as DNMTs catalyse the insertion of methyl groups into DNA, and they are essential for the maintenance of aberrant methylation in cancer cells. Exhibitors that have been demonstrated to reverse DNA hyper methylation and restore the expression of silenced tumour suppressor genes include 5-azacytidine (Azacitidine) and decitabine (5-aza-2'-deoxycytidine). By turning these vital genes back on, DNMT inhibitors have shown in preclinical models of oral cancer that they can slow the growth of tumours. To find out how safe and effective these medications are for patients with oral cancer, clinical trials are currently being conducted [20].

Proteins called histones are the building blocks of chromatin, which encases DNA and forms the skeleton of chromosomes. Histone modifications, including acetylation, methylation, and phosphorylation, affect the degree to which DNA is coiled around these proteins, controlling the expression of genes. The dysregulation of genes involved in cell cycle control, apoptosis, and DNA repair can be caused by aberrant histone modifications in oral cancer. Active transcription of genes is linked to histone acetylation, whereas deacetylation tends to repress gene activity. Tumour suppressor genes may be silenced or oncogenes may be activated because of changes in histone modification patterns in oral cancer, which can accelerate the disease's growth [21].

Gene repression and a more compact chromatin structure are the results of histone deacetylases (HDACs) removing acetyl groups from histones. Tumour suppressor genes are silenced in oral cancer because HDACs are frequently overexpressed in this disease. Histone acetylation can be restored by HDAC inhibitors (HDACis), which reactivates genes that have been silenced. This makes HDACis a promising treatment option for oral cancer. By reactivating tumour suppressor genes and sensitizing cancer cells to chemotherapy and radiation, medications like vorinostat (SAHA) and romidepsin have shown promise in preclinical studies. HDAC inhibitors provide a new way to improve treatment responses for patients with oral cancer by reversing epigenetic silencing [22].

**Fig. 5:**
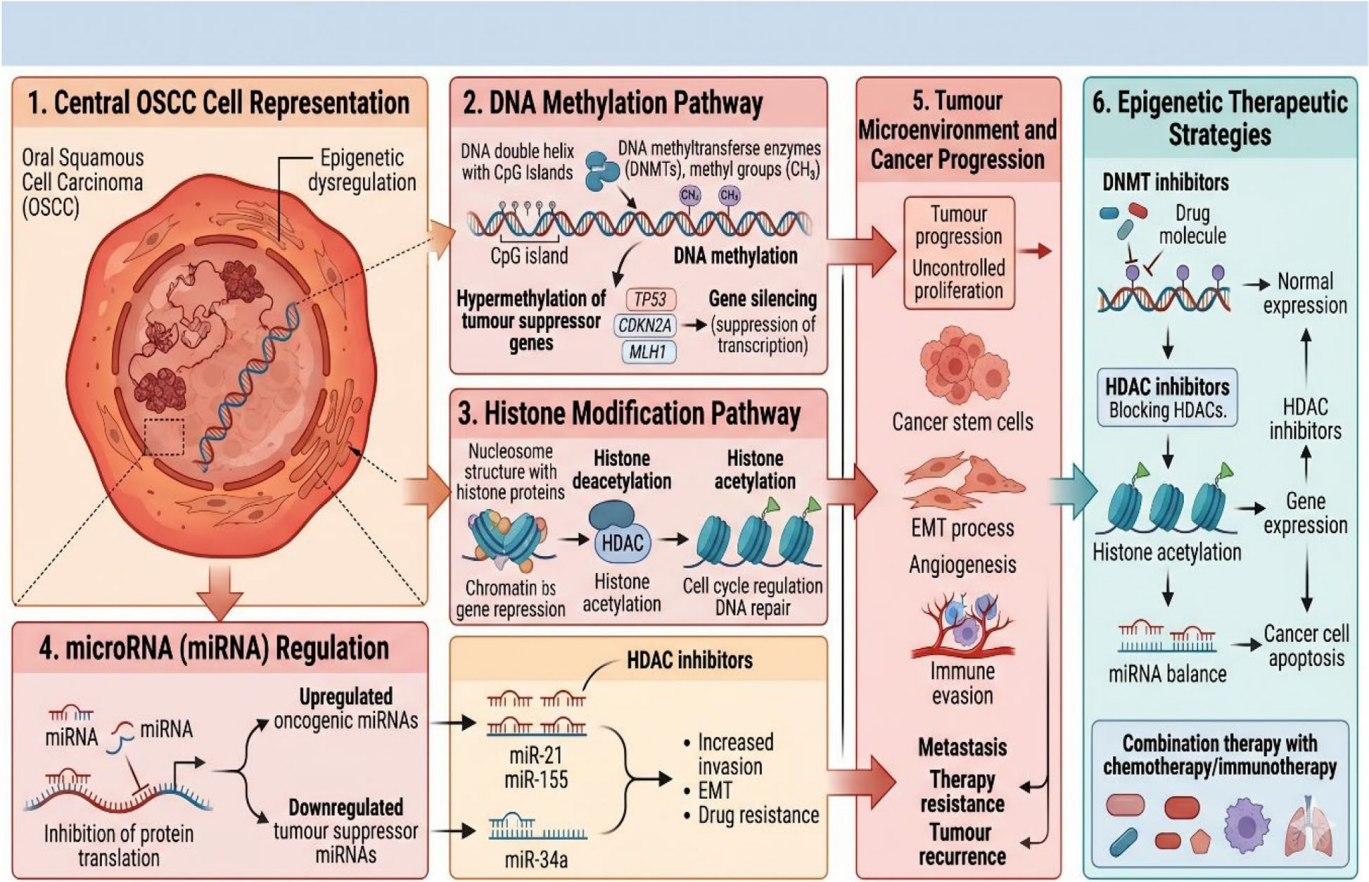
Epigenetic modifications in OSCC

Combining DNMT and HDAC inhibitors with traditional cancer treatments like chemotherapy and radiation therapy is one of the most exciting advances in epigenetic therapy. Treatment sensitivity of a tumour can be influenced by epigenetic changes. For example, epigenetic drugs could reactivate silenced genes involved in DNA repair or apoptosis, increasing the susceptibility of cancer cells to agents that damage DNA, such as radiation or cisplatin. To increase treatment efficacy, decrease tumour resistance, and enhance patient outcomes, clinical trials are investigating the effectiveness of combining DNMT or HDAC inhibitors with conventional oral cancer therapies [23]. Epigenetic Modifications in Oral Squamous Cell Carcinoma are summarized in Fig. 5. MicroRNAs, which are small non-coding RNA molecules, control the expression of genes by directing messenger RNA (mRNA) towards translational repression or degradation. Certain miRNAs function as tumour suppressors or oncogenes in oral cancer and are controlled by epigenetic processes like histone modification and DNA methylation. For instance, silencing of tumour suppressor miRNAs due to hyper methylation of their promoter regions can aid in the development of cancer. On the other hand, epigenetic modifications that encourage the growth of cancer can cause oncogenic miRNAs to be overexpressed. Comprehending the relationship between miRNAs and epigenetic modifications in oral cancer could result in the creation of innovative treatment approaches focused on reestablishing typical miRNA expression patterns [24]. Techniques and mechanisms related to tissue engineering advancements in oral cancer are summarised (Table 2).

**Table 2:** Techniques and mechanisms related to tissue engineering advancements in oral cancer

Technique	Mechanism	Application in Oral Cancer
Scaffold Fabrication	Provides structural support for cell attachment, growth, and differentiation	Bone and soft tissue regeneration post-tumour resection
3D Bio printing	Layer-by-layer deposition of bio-inks containing cells and biomaterials to create custom constructs	Development of patient-specific scaffolds for tissue replacement
Hydrogel-Based Models	Mimics extracellular matrix (ECM) to create a biocompatible environment for cell growth	Modelling tumour microenvironment for drug screening
Stem Cell Therapy	Differentiation of stem cells into specific tissue types through signalling molecules and growth factors	Regeneration of oral mucosa, bone, and vascular tissues
Gene Therapy	Delivery of therapeutic genes to modify or silence oncogenes and promote tissue repair	CRISPR-Cas9 for cancer gene editing, enhancing cell survival in engineered tissues
Decellularization	Removal of cellular components from donor tissues while preserving ECM structure	Creating biocompatible scaffolds for oral tissue reconstruction
Nanotechnology	Integration of nanoparticles to enhance scaffold properties and drug delivery	Targeted drug delivery, improved scaffold mechanical strength
Microfluidic Systems	Simulates dynamic conditions like nutrient flow and shear stress	Testing tumour response to therapies in a controlled microenvironment
Bioactive Molecules	Incorporation of growth factors, cytokines, and chemokines to stimulate cell proliferation	Accelerated healing and improved integration of engineered tissues
Organoid Culture	Self-organizing 3D cell culture systems that mimic the functionality of tissues	Studying oral cancer biology and testing personalized treatments

## Challenges associated with recent approaches

Despite significant advancements in tissue engineering and precision therapeutics for OSCC,

Several clinical, technical, ethical, and translational challenges continue to limit their.

Widespread clinical application.

### Donor site morbidity

Complications include discomfort, scarring, and functional limitations may arise after tissue harvesting from donor locations [83].

### Surgical complexity

Autologous tissue harvesting and transfer is a difficult process that calls for sophisticated surgical methods, raising the possibility of problems [84].

Although CRISPR presents previously unheard-of chances to advance research on oral cancer, it also poses significant ethical issues. Off-target effects, or the editing of unwanted genes, continue to be a worry, particularly in clinical applications [18]. Furthermore, there has been a great deal of discussion around the potential use of CRISPR for germline editing, which would transfer genetic modifications to subsequent generations. Inspite of these obstacles, CRISPR has a promising future in the study of oral cancer. CRISPR technology is still evolving, and new developments like base editors—more accurate gene-editing instruments—are probably going to make CRISPR an even more potent weapon in the fight against oral cancer. One day, with more research and development, CRISPR may result in individualized therapies that significantly enhance patient outcome.

Even though epigenetic therapies have a lot of potential to treat oral cancer, there are still some obstacles to overcome. The specificity of these treatments is one of the main issues since broad-acting epigenetic medications may alter the expression of genes in both healthy and malignant cells, which could result in undesirable side effects. The goal of ongoing research is to create more precisely targeted epigenetic treatments that alter the epigenome of cancer while sparing healthy tissues. Furthermore, additional clinical trials are required to evaluate the long-term safety and effectiveness of these treatments for patients with oral cancer. Notwithstanding these obstacles, epigenetic therapy appears to have a bright future in the treatment of oral cancer. It may supplement current therapies and give patients new hope [19].

New targets for therapeutic intervention are provided by epigenetic changes, which are crucial in the initiation and spread of oral cancer. Treatment for oral cancer is being investigated through several mechanisms, including DNA methylation, histone modification, and miRNA regulation. Improving the efficacy of current treatments and reversing aberrant gene silencing are possible with the use of DNMT inhibitors, HDAC inhibitors, and other epigenetic modulators. To improve patient outcomes and survival rates, epigenetic therapies may play a significant role in the individualized treatment of oral cancer as research progresses [129].

## Integrated precision regenerative strategies for oral cancer therapy

Recent advances in tissue engineering have shifted the focus of oral squamous cell carcinoma (OSCC) therapy from isolated regenerative approaches toward integrated and multifunctional therapeutic platforms capable of simultaneously supporting tissue regeneration, targeted drug delivery, immunomodulation, and precision oncology applications [130]. Emerging evidence suggests that synergistic integration of biomaterials, stem cell engineering, 3D bio printing, nanotechnology, epigenetic modulation, and CRISPR/Cas9-mediated gene editing may significantly enhance therapeutic efficacy and regenerative outcomes in oral cancer management. Such interdisciplinary convergence represents a pivotal step toward the development of personalized regenerative oncology strategies capable of overcoming the limitations associated with conventional therapies [131]. Among these emerging technologies, 3D bio printing has demonstrated substantial potential for fabricating biomimetic scaffolds that closely replicate the structural and biological complexity of the oral tumour microenvironment. Advanced 3D bio printed constructs enable precise spatial distribution of cells, extracellular matrix components, growth factors, and bioactive molecules, thereby facilitating the development of highly controlled regenerative microenvironments [132]. Furthermore, multifunctional bio printed scaffolds may serve as localized platforms for temporally regulated delivery of CRISPR/Cas9 systems, RNA therapeutics, stem cells, and chemotherapeutic agents, thereby improving targeting precision while minimizing systemic toxicity [133]. Recent studies have demonstrated that 3D bio printed tumour models can effectively recapitulate tumour heterogeneity, stromal interactions, angiogenesis, and immune-cell dynamics, thereby enhancing personalized therapeutic design and drug-screening applications in OSCC [134]. The integration of nanotechnology with tissue-engineered scaffolds has further accelerated the development of multifunctional regenerative systems. Nanostructured biomaterials such as halloysite nanotubes, functionalized Nano diamonds, and hybrid bio inks possess unique physicochemical properties including high surface area, tunable surface chemistry, enhanced mechanical stability, and controlled release behaviour [135]. These nanomaterials can function as scaffold-integrated Nano carriers capable of delivering growth factors, nucleic acids, anti-cancer drugs, and immunomodulatory molecules directly to tumour or regenerative sites [37]. Incorporation of nanomaterials into bio inks additionally improves scaffold printability, cellular adhesion, angiogenesis, and long-term tissue integration, thereby enhancing the regenerative performance of 3D bio printed oral tissue constructs [131]. Recent evidence also suggests that epigenetic reprogramming strategies may significantly enhance the regenerative functionality of stem cell-based therapies in oral oncology. Epigenetic modulation of stem cells through regulation of DNA methylation, histone modification, and non-coding RNA signalling has demonstrated the potential to improve stem cell homing, differentiation fidelity, immunomodulatory behaviour, and tissue-regenerative capacity [135]. Furthermore, epigenetic regulation may suppress tumour-promoting inflammatory pathways and enhance tumour-suppressive activity within the oral tumour microenvironment. Integration of epigenetic therapies with stem cell-loaded scaffolds and biomaterial-based delivery systems may therefore provide highly targeted and regenerative therapeutic platforms for OSCC reconstruction and management [136]. Artificial intelligence (AI)-assisted biomaterial engineering and computational modelling are also expected to play increasingly important roles in the future of integrated oral cancer therapy [130]. AI-driven platforms may optimize scaffold architecture, biomaterial composition, drug-release kinetics, and patient-specific regenerative design by analysing tumour heterogeneity, molecular biomarkers, and tissue micro environmental characteristics. Such technologies may facilitate the fabrication of personalized scaffolds capable of simultaneously supporting angiogenesis, immune modulation, controlled therapeutic release, and tissue regeneration [137]. Collectively, the convergence of biomaterials science, regenerative medicine, nanotechnology, gene editing, epigenetic engineering, and 3D bio printing represents a transformative paradigm in oral cancer therapy [138]. Future multifunctional scaffold systems are anticipated to function not only as structural regenerative matrices but also as intelligent therapeutic platforms capable of coordinating tissue regeneration, localized drug delivery, immune regulation, and precision molecular targeting within the OSCC microenvironment [131]. Continued interdisciplinary research and translational optimization will be essential to facilitate clinical implementation of these integrated regenerative oncology strategies [138].

## Future outlook

Research in the rapidly expanding field of epigenetic biomarker identification for oral cancer. Epigenetic modifications have a lot of potential as diagnostic tools for early detection because they frequently take place early in the development of cancer. For example, saliva and tissues from patients with oral cancer have been shown to have hyper methylation of genes, such as RASSF1A or DAPK. Through non-invasive testing, these epigenetic biomarkers can be found, offering a way to identify oral cancer early on when it is more manageable. With further research, epigenetic biomarkers could play a crucial role in individualized cancer care by monitoring the course of the disease and guiding treatment choices [139]. One significant obstacle in the treatment of oral cancer is drug resistance. Many tumours respond to radiation or chemotherapy at first, but as time goes on, they become resistant and relapse. It is thought that epigenetic changes contribute to the emergence of this resistance. For instance, cancer cells may continue to grow despite therapy if pro-apoptotic genes are silenced. [140] Drug resistance is being studied as a potential solution using epigenetic therapies, such as DNMT and HDAC inhibitors. These treatments can increase treatment responsiveness in resistant cancer cells by reactivating silenced genes linked to cell death pathways, which may enhance long-term survival results [141]. Through genetic engineering or medication, cancer cells' epigenetic makeup can be reset, effectively "reprogramming" them to become less malignant. This process is known as epigenetic reprogramming. Researchers are looking into how epigenetic reprogramming might be able to stop cells from acting cancerous in cases of oral cancer [142]. This strategy might use DNMT inhibitors, HDAC inhibitors, and other epigenetic medications to target several epigenetic modifications at once. Through modifications to the expression of essential genes involved in DNA repair, apoptosis, and cell cycle regulation, epigenetic reprogramming may offer a novel therapeutic approach to address the underlying causes of oral cancer [17]. Stem cell-based regenerative medicine is expected to revolutionize personalized oral and maxillofacial reconstruction. Mesenchymal stem cells (MSCs), oral stem cells, induced pluripotent stem cells (iPSCs), and urine-derived stem cells (UDSCs) possess remarkable regenerative, immunomodulatory, and multilineage differentiation capabilities that may facilitate the restoration of both hard and soft oral tissues following oncologic treatment [2, 8, 9]. In particular, combining stem cells with biomimetic hydrogels, nanostructured scaffolds, and 3D bio printed constructs is anticipated to improve cellular retention, vascularization, tissue integration, and long-term regenerative outcomes [9]. Personalized regenerative platforms fabricated through 3D bio printing technologies may additionally enable the development of patient-specific tissue constructs that closely mimic the native oral microenvironment. Emerging gene-editing technologies such as CRISPR/Cas9 are expected to introduce transformative opportunities in precision oral oncology. These technologies may enable selective correction of oncogenic mutations, targeted elimination of cancer stem cells, modulation of tumor-associated signaling pathways, and reversal of therapeutic resistance. Integration of CRISPR systems with nanoparticle-mediated delivery platforms may further improve targeting specificity while minimizing systemic adverse effects [4]. In parallel, epigenetic therapies targeting DNA methylation, histone modifications, and non-coding RNAs may provide novel therapeutic avenues for suppressing tumor progression and preventing recurrence [4]. Artificial intelligence (AI) and machine learning technologies are also anticipated to significantly accelerate progress in oral cancer diagnosis, biomaterial design, therapeutic optimization, and personalized treatment planning. AI-assisted platforms may facilitate precise analysis of tumor heterogeneity, molecular biomarkers, and treatment responses, thereby improving clinical decision-making and therapeutic outcomes [4]. Furthermore, bioengineered exosomes and plant-derived extracellular vesicles have emerged as highly promising Nano therapeutic systems because of their excellent biocompatibility, low immunogenicity, and targeted drug delivery capabilities. These naturally derived Nano vesicles may effectively transport chemotherapeutic agents, nucleic acids, and immunomodulatory molecules to tumor tissues while modulating the tumor microenvironment and reducing systemic toxicity [4]. Although significant challenges related to clinical translation, regulatory approval, biosafety validation, and large-scale manufacturing remain, continued multidisciplinary collaboration among clinicians, biomaterial scientists, molecular biologists, and bioengineers is expected to accelerate the development of clinically applicable regenerative therapies for oral cancer. Collectively, the integration of tissue engineering, nanotechnology, stem cell biology, and precision medicine holds immense promise for establishing safer, more effective, and highly personalized therapeutic strategies for the future management of OSCC.

## Conclusion

Treating oral cancer with tissue engineering offers novel approaches to tissue regeneration and recovery from cancer therapy, making it a promising new area of research. Improved and individualized treatments for oral cancer are becoming possible thanks to developments in 3D bio printing, stem cell therapy, and scaffold design. The use of autologous tissue, chip models, and CRISPR and gene editing has contributed to the advancement of tissue engineering for oral cancer the potential of technology is significant in improving therapeutic strategies and patient outcomes. Biocompatibility is improved and rejection rates are reduced using autologous tissue, and chip models offer a powerful platform for studying the behaviour and response of tumours in a controlled setting. Moreover, precision can be achieved using CRISPR and gene editing technologies when it comes to modifying cancer cell lines and Aiding in a better comprehension of the genetic underpinnings of oral cancer and advancing targeted therapies. By combining these innovations, a multidisciplinary approach can emerge that can lead to more effective and individualized treatments for patients who are suffering from oral cancer. The development of these ground-breaking treatments and the enhancement of oral cancer patients' quality of life will depend on ongoing research and cooperation amongst bioengineers, oncologists, and clinicians—even in the face of ongoing obstacles.

## Data Availability

No datasets were generated or analysed during the current study.
